# *Apium graveolens*-associated *Aspergillus* sp.: metabolomic profiling and anti-MRSA potential supported by in silico studies

**DOI:** 10.1186/s12934-025-02645-9

**Published:** 2025-03-08

**Authors:** Alshymaa Abdel-Rahman Gomaa, Hesham A. Abou-Zied, Sara Mahmoud Farhan, Ruqaiah I. Bedaiwi, Mohammad A. Alanazi, Stefanie P. Glaeser, Peter Kämpfer, Usama Ramadan Abdelmohsen, Fatma Alzahraa Mokhtar, Enas Reda Abdelaleem

**Affiliations:** 1 Department of Pharmacognosy, Faculty of Pharmacy, Minia University, Minia, 61519 Egypt; 2 Department of Medicinal Chemistry, Faculty of Pharmacy, Deraya University, Minia, 61111 Egypt; 3 Department of Microbiology and Immunology, Faculty of Pharmacy, Deraya University, Minia, 61111 Egypt; 4 Department of Medical Laboratory Technology, Faculty of Applied Medical Sciences, University of Tabuk, 71491 Tabuk, Saudi Arabia; 5 Institute of Applied Microbiology, Justus-Liebig University Gießen, Gießen, Germany; 6 Deraya Center for Scientific Research, Deraya University, Minia, 61111 Egypt; 7 Department of Pharmacognosy, Faculty of Pharmacy, El Saleheya El Gadida University, El Saleheya El Gadidam, 44813 Sharkia Egypt; 8 Fujairah Rsearch Centre, Sakamkam Road, Sakamkam, Fujairah, 00000 UAE

**Keywords:** Anti-MRSA, *Aspergillus*, *Apium graveolens*, In silico

## Abstract

Methicillin-resistant *Staphylococcus aureus* (MRSA) is a significant pathogen associated with healthcare-related infections that are often challenging to treat. Conditions such as, skin and soft tissue infections, bloodstream infections, and pneumonia highlight the critical need for effective therapeutic strategies. Careful use of antibiotics under medical supervision is essential to prevent the further emergence of MRSA. Recent studies have documented the antibacterial efficacy of certain endophytic fungi extracts against MRSA, suggesting their potential as a source of novel treatments. This study investigates the metabolomic profiling of the endophytic fungus *Aspergillus* sp. SH1 using liquid chromatography-high-resolution electrospray ionization mass spectrometry (LC-HR-ESI-MS) and evaluates the anti-MRSA potential of the fungal extract. The metabolomic analysis identified 27 compounds (**1–27**) with diverse chemical natures, including polyketides, alkaloids, cyclic tripeptides, polypropionate derivatives, and sesquiterpenes. The fungal extract exhibited potent anti-MRSA activity, with an IC_*50*_ value of 9.8 µg/mL, compared to ciprofloxacin (IC_*50*_ = 25.7 µg/mL). To support these findings, in silico studies were performed to model the binding interactions of the identified compounds with key MRSA-related targets, including Toll-like receptor 2 (TLR2), von Willebrand factor (VWF), tumor necrosis factor (TNF), and penicillin-binding protein 2a (PBP2a). Compounds **2, 9, 15, 16, 20, 22,** and **25** demonstrated enhanced binding affinities, suggesting their potential as lead molecules for developing new antibacterial agents targeting MRSA. In conclusion, this study highlights the promising anti-MRSA potential of *Aspergillus* sp. SH1 extract, providing a foundation for further exploration of its bioactive compounds in combating resistant bacterial infections.

**Supplementary Information:**

The online version contains supplementary material available at 10.1186/s12934-025-02645-9.

## Introduction

Plant endophytes, including bacteria and fungi, inhabit the healthy tissues of their host plants during all or part of their life cycle without causing visible symptoms of disease. These microorganisms are a rich and intriguing source of bioactive secondary metabolites, capable of producing unique constituents that may either resemble the plant's own compounds or be entirely novel [1]. The relationship between endophytes and their host plants is often mutualistic; the plant provides nutrition and protection, while the endophyte produces bioactive substances that enhance the plant's growth and survival under various environmental stresses [2].

The bioactive compounds produced by endophytic fungi have garnered attention for their potential applications in medicine and agriculture, particularly in combating antimicrobial resistance (AMR). With the rise of multidrug-resistant microorganisms, including *Enterococci*, *Streptococci*, *Gonococci*, and *Staphylococci*, there is an urgent need to discover innovative chemicals for therapeutic purposes [3]. One of the most pressing examples is methicillin-resistant *Staphylococcus aureus* (MRSA), a bacterial strain that has developed resistance to multiple antibiotics, posing a significant threat to public health [4, 5].

In this context, the endophytic fungi associated with plants represent a promising avenue for the discovery of novel antimicrobial agents. This study investigates the metabolomic profiling of the endophytic fungus *Aspergillus* sp. SH1 and evaluates its anti-MRSA potential through both in vitro and in silico methods. These findings aim to advance our understanding of endophytic fungi as sources of bioactive compounds and their role in addressing the global AMR crisis.

Thus, discovering novel antibiotics is essential to fight these resistant microorganisms. Endophytic fungus' natural components could be used as novel antibiotics to combat harmful bacteria [6]. Consequently, the endophytic fungi of medicinal plants have received great attention in recent years. These plants are considered a significant and potential source for the discovery of microorganisms as well as a rich supply of innovative and beneficial natural metabolites of interest to pharmaceutical and agricultural potential [7]. Hence, there are several instances of endophytes producing secondary metabolites that have potential applications in medicine or agriculture. Guanacastepenes, for instance, which was produced by an unidentified endophytic fungus that was isolated from the *Daphnopsis americana* tree, has shown strong antibacterial action against *Enterococcus faecium* and drug-resistant *Staphylococcus aureus.* In addition, cryptocin is produced by the endophytic *Cryptosporiopsis quercina* and it exhibited strong anti-*Pyricularia oryzae* activity [8, 9].

One of the most well-known filamentous fungi in the Ascomycetes family is *Aspergillus*. These fungi are aerobics and can thrive in conditions rich in oxygen, and many of them can even grow in environments devoid of essential nutrients. They exist in nature as parasites, saprophytes, endophytes, and human pathogens [10, 11]. Numerous studies have reported that endophytic *Aspergillus* species are abundant and renewable sources of bioactive secondary metabolites, including alkaloids, terpenoids, cytochalasins, sterols, and xanthones, which are crucial to the pharmaceutical and commercial industries. The secondary metabolites produced by different endophytic *Aspergillus* species exhibited several biological actions, including anti-inflammatory, anti-cancer, anti-bacterial, and anti-viral properties [12]. For instance, endolichenic fungi (ELF) represent a prolific source of structurally diverse bioactive metabolites, including alkaloids, quinones, and terpenes, with significant biological activities such as anticancer and antimicrobial effects. Approximately 100 novel compounds were identified among 172 metabolites isolated between 2008 and 2019, highlighting ELF's potential in drug discovery and the activation of silent biosynthetic gene clusters. Notably, several ELF-derived compounds exhibit potent anticancer properties, demonstrating mechanisms such as caspase-3 inhibition, induction of G0/G1 cell cycle arrest, and promotion of apoptosis [13].

Similarly, mangrove endophytic fungi, which thrive under extreme environmental conditions, are also a promising source of bioactive metabolites with diverse chemical structures, such as peptides, alkaloids, and glycosides. These metabolites display potent antibacterial, antifungal, antiviral, and antimycobacterial activities, further emphasizing the importance of natural products from endophytic fungi in combating drug-resistant pathogens [14]. Moreover, endophytic fungi are gaining attention as a source of bioactive metabolites for treating diabetes mellitus, a rapidly growing global health concern [15]. The significance of endophytic fungi in providing novel bioactive compounds for breast cancer therapy is also increasingly recognized. Following the discovery of taxol, numerous endophytic fungal metabolites with potential anticancer activity, particularly against breast cancer, have been identified, though most have yet to progress to clinical trials. Continued research, incorporating advanced technologies such as multi-omics and pharmacological tools, could unlock their full therapeutic potential, leading to the development of low-cost, highly specific, and less toxic chemotherapeutic agents from endophytic fungi [16].

*Staphylococcus aureus* is a Gram-positive coccoid bacterium, and it is considered an opportunistic microorganism that attacks open wounds and individuals with a weakened immune system. Methicillin is a semisynthetic penicillin capable of penetrating bacterial cell walls. The resistance to methicillin by *S. aureus* was initially observed in 1961, shortly after the antibiotic was introduced clinically. Subsequently, there has been a global epidemic of MRSA in both healthcare and community settings [17, 18].

MRSA is a major nosocomial pathogen that has emerged from a hospital setting and is responsible for many infections associated with health care that are usually difficult to treat. MRSA exhibits two common resistance mechanisms, including overexpression of *β*-lactamases and a change in the normal structure of penicillin-binding proteins (PBPs) [19, 20]. Currently, vancomycin and daptomycin are used to treat severe MRSA infections. They are also used to treat complex skin and soft tissue infections, hospital-associated pneumonia, and bacteremia. However, the abuse of vancomycin in hospitals has resulted in vancomycin resistant MRSA, while daptomycin is still useful, but its expense is restricting its application [21, 22]. Consequently, it is necessary to use antibiotics carefully and under medical supervision to prevent an increased MRSA risk. Recently, several studies have reported the antimicrobial efficacy of various endophytic fungal extracts against MRSA. Thus, these endophytic fungi may have the potential to be effective options for treating MRSA infections.

This study involved the isolation and identification of endophytic fungus from *Apium graveolens* L. (F. Apiaceae) seeds, that was being grown in Egypt, was performed. The isolated fungus was determined to be an *Aspergillus* sp. based on morphological, microscopic, and molecular biological identification. Additionally, the anti-MRSA potential of this fungal strain was evaluated. Furthermore, LC-HR-ESI-MS based metabolomics was used to investigate the chemical profile of the fungal extract, followed by in silico molecular docking study.

## Result and discussion

### Identification and phylogenetic diversity of fungus associated with *A. graveolens* seeds

Phylogenetic analyses based on the internal transcribed spacer region (ITS 1 and 2) (Figure S1) and partial 18S rRNA gene sequence (Figure S2) placed the fungal strain SH1 into the *Aspergillus* section *niger* group [23]*.* The strain shared an identical ITS sequence with *A. neoniger* CBS 115656 (NR_137505.1) and 99.5–99.8% ITS sequence similarity to the type material of other species in the *Aspergillus* section *niger group.* The next related 18S rRNA gene sequence was that of *A. niger* CBS 554.65 (NG 065763.1) with 99.9% sequence similarity; however, an 18S rRNA gene sequence of *A. neoniger* was not in that database.

### Metabolomic analysis of the total extract of *Aspergillus* associated with *A. graveolens* seeds

**Fig. 1:**
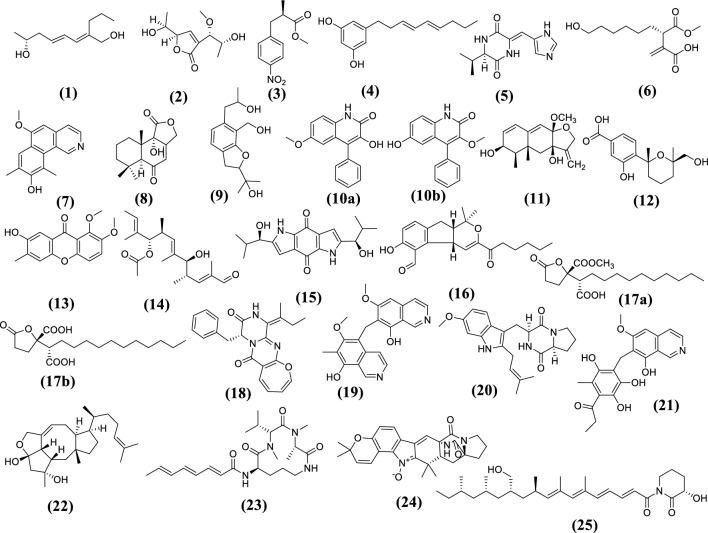
Chemical structures of the tentatively identified metabolites from *Aspergillus* sp.

Metabolomic analysis of the crude extract of *Aspergillus* sp. associated with *A. graveolens* seeds using high resolution electrospray ionization mass spectroscopy (HR-ESI-MS) (Figure S3 and S4) resulted in the identification of a variety of metabolites with different chemical natures (Fig. 1; Table 1). The mass ion peak at *m/z* 183.1381 [M−H]^−^ was in agreement with the molecular formula C_11_H_20_O_2_. It was dereplicated as the hydroxylated hydrocarbon aspergone M **(1)**, which was previously isolated from *Aspergillus* sp. OUCMDZ-1583 [24]. Another mass ion peak at *m/z* 217.1063 [M+H]^+^ was corresponding to the molecular formula C_10_H_16_O_5_. It was determined to be a polyketide aspilactonol D **(2)**, which was previously isolated from *Aspergillus* sp. 16-02-1[25]. Similarly, the mass ion peak at *m/z* 222.0794 [M−H]^−^ was annotated as methyl α-methyl-4-nitrobenzenepropanoate **(3)** in agreement with the molecular formula C_11_H_13_NO_4_. This nitrogenous phenylpropionic acid derivative was previously reported in *A. terreus* LPFH-1 [26]. Whereas that at *m/z* 233.1491 [M+H]^+^ was corresponding to the molecular formula C_15_H_20_O_2_, which was matched with the phenolic compound 5-[(3*E*,5*E*)-3,5-nonadienyl]-1,3-benzenediol **(4)** and was previously isolated from *Aspergillus* sp. [27].

**Table 1:** Results of metabolomics analysis of *Aspergillus* sp. extract

N	Compound	Accurate mass	Mode	m/z	Rt	Structure	Source	Bioactivity	Reference
1	Aspergone M C_11_H_20_O_2_	184.1463	−	183.1381	4.04		*Aspergillus* sp. OUCMDZ-1583	Not detected	[24]
2	Aspilactonol D C_10_H_16_O_5_	216.0998	+	217.1063	1.81		*Aspergillus* sp. 16-02-1	Not detected	[25]
3	Methyl *α*-methyl-4-nitrobenzenepropanoate C_11_H_13_NO_4_	223.0845	−	222.0794	2.32		*Aspergillus terreus* LPFH-1	Not detected	[26]
4	5-[(3*E*,5*E*)-3,5-Nonadienyl]-1,3-benzenediol C_15_H_20_O_2_	232.1463	+	233.1491	2.04		*Aspergillus* sp.	Not detected	[27]
5	JBIR-75 C_11_H_14_N_4_O_2_	234.1117	+	235.1186	1.67		*Aspergillus* sp. fS14	Not detected	[28]
6	Asperitaconic acid A C_12_H_20_O_5_	244.1311	−	243.1295	2.65		*Aspergillus niger*	Active against* Staphylococcus aureus*	[29]
7	Puniceusine G C_16_H_15_NO_2_	253.1103	+	254.1183	2.21		*Aspergillus puniceus* SCSIO z021	Active against *S. aureus* and MRSA	[30]
8	9*α*-Hydroxy-5*α*-drim-7-ene-6-one-11,12-olide C_15_H_20_O_4_	264.1361	−	263.1277	3.82		*Aspergillus carneus* KMM 4638	Active against *S. aureus* and *Bacillus cereus*	[31]
9	Ustusorane E C_15_H_22_O_4_	266.1518	−	265.1476	4.68		*Aspergillus ustus* 094102	Not detected	[32]
10a	3-Hydroxy-6-methoxy-4-phenylquinolin-2(1*H*)-one C_16_H_13_NO_3_	267.0895	−	266.0818	2.79		*Aspergillus versicolor* AS-212	Not detected	[33]
10b	3-Methoxy-6-hydroxy-4-phenylquinolin-2(1*H*)-one C_16_H_13_NO_3_							Not detected	
11	Dihydrobipolaroxin B C_16_H_22_O_4_	278.1518	−	277.1448	5.53		*Aspergillus* sp. SCSIOW2	Not detected	[34]
12	3-Hydroxy4-((2 *R*,6 *R*)-6-(hydroxymethyl)-2,6-dimethyltetrahydro-2 *H*-pyran2-yl) benzoic acid C_15_H_20_O_5_	280.1311	−	279.1222	2.99		*Aspergillus *sp*.* SCSIO06786	Not detected	[35]
13	Versicone J C_16_H_14_O_5_	286.0841	−	285.0791	3.21		*Aspergillus versicolor* D5	Not detected	[36]
14	Aspormisin A C_19_H_30_O_4_	322.2144	−	321.2096	6.11		*Aspergillus ochraceopetaliformis* SCSIO 41020	Not detected	[37]
15	Terreusinone C_18_H_22_N_2_O_4_	330.1579	−	329.1508	1.92		*Aspergillus terreus*	Not detected	[38]
16	Aspergillone C_21_H_26_O_4_	342.1831	−	341.1782	3.91		*Aspergillus versicolor*	Not detected	[39]
17a	Spiculisporic acid C C_18_H_30_O_6_	342.2042	−	341.1973	5.37		*Aspergillus* sp. HDf2	antibacterial activities against *S. aureus*	[40]
17b	Spiculisporic acid D C_18_H_30_O_6_							antibacterial activities against *S. aureus*	
18	Protuboxepin G C_22_H_21_N_3_O_3_	375.1583	−	374.1471	1.85		*Aspergillus versicolor* SCSIO 41016	Not detected	[41]
19	Puniceusine E C_22_H_20_N_2_O_4_	376.1423	+	377.1475	2.52		*Aspergillus puniceus* SCSIO z021	Not detected	[30]
20	Tryprostatin A C_22_H_27_N_3_O_3_	381.2052	+	382.2142	5.16		*Aspergillus fumigatus*	Not detected	[42]
21	Puniceusine J C_21_H_21_NO_6_	383.1369	−	382.1251	1.80		*Aspergillus puniceus* SCSIO z021	Not detected	[30]
22	(5*S*,6*S*)-16,17-Dihydroophiobolin H C_25_H_40_O_3_	388.2977	+	389.3047	5.36		*Aspergillus insuetus* SD-512	Not detected	[43]
23	Sclerotiotide E C_23_H_36_N_4_O_4_	432.2736	+	433.2806	4.68		*Aspergillus sclerotiorum* PT06-1	Not detected	[44]
24	6-*epi*-Avrainvillamide C_26_H_27_N_3_O_4_	445.2001	−	444.1955	2.60		*Aspergillus taichungensis*	Not detected	[45]
25	Fiscpropionate D C_29_H_47_NO_4_	473.3505	−	472.346	6.22		*Aspergillus fischeri* FS452	Not detected	[46]

Besides, the mass ion peak at *m/z* 235.1186 [M+H]^+^ was in agreement with the molecular formula C_11_H_14_N_4_O_2_. It was annotated as the roquefortine analogue JBIR-75 **(5)** [28]. Asperitaconic acid A **(6)** was identified depending on the mass ion peak at *m/z* 243.1295 [M−H]^−^ and in agreement with the molecular formula C_12_H_20_O_5_. Asperitaconic acid A was previously isolated from *A. niger* and exhibited antibacterial effect against *Staphylococcus aureus* [29]. Similarly, the alkaloid puniceusine G **(7)** was identified depending on the mass ion peak at *m/z* 254.1183 [M+H]^+^, which was in agreement with the molecular formula C_16_H_15_NO_2_. It was previously reported from the marine derived fungus *A. puniceus* SCSIO z021 [30]. Additionally, the mass ion peak at *m/z* 263.1277 [M−H]^−^ was dereplicated as the drimane sesquiterpenoid, 9*α*-hydroxy-5*α*-drim-7-ene-6-one-11,12-olide **(8)**, in agreement with the molecular formula C_15_H_20_O_4_ and was previously isolated from *A. carneus* KMM 4638 [31]. The isochromane derivative, ustusorane E **(9)**, was identified according to the mass ion peak at *m/z* 265.1476 [M−H]^−^ and in match with the molecular formula C_15_H_22_O_4_. Ustusorane E was reported in *A. ustus* 094102 [32]. The mass ion peak at *m/z* 266.0818 [M−H]^−^ was identified according to the molecular formula C_16_H_13_NO_3_ to be either 3-hydroxy-6-methoxy-4-phenylquinolin-2(1*H*)-one **(10a)** or 3-methoxy-6-hydroxy-4-phenylquinolin-2(1*H*)-one **(10b)**. The quinolinone alkaloid analogs **10a** and **10b** were isolated from the deep sea derived fungus *A. versicolor* AS-212 and exhibited antibacterial activity against the aquatic pathogenic micro-organisms, *Vibrio harveyi* and *V. alginolyticus* and displayed antifungal activities against plant pathogenic micro-organisms *Epicoccum sorghinum*, *Colletotrichum gloeosporioides*, and *Curvularia spicifera* [33].

In addition, the mass ion peak at *m/z* 277.1448 [M−H]^−^ was dereplicated as the sesquiterpenoid dihydrobipolaroxin B **(11)** in agreement with the molecular formula C_16_H_22_O_4_ and was reported in the deep marine-derived fungus, *Aspergillus* sp. SCSIOW2 [34]. Another mass ion peak at *m/z* 279.1222 [M−H]^−^ was annotated as 3-hydroxy4-((2 *R*,6 *R*)-6-(hydroxymethyl)-2,6-dimethyltetrahydro-2 *H*-pyran2-yl) benzoic acid **(12)** in agreement with the molecular formula C_15_H_20_O_5_ and was previously isolated from *Aspergillus* sp. SCSIO06786 [35]. Further, the mass ion peak at *m/z* 285.0791 [M−H]^−^ was designed to be versicone J **(13)** according to the molecular formula C_16_H_14_O_5_.This xanthone derivative was isolated from the alga-derived fungus *A. versicolor* D5 [36]. Moreover, the polyketide, aspormisin A **(14)**, was identified in agreement with the molecular formula C_19_H_30_O_4_ and previously reported from the alga-derived fungus *A. ochraceopetaliformis* SCSIO 41020 [37]. Similarly, terreusinone **(15)** was in agreement with the mass ion peak at *m/z* 329.1508 [M−H]^−^ and the molecular formula C_18_H_22_N_2_O_4_. It was previously isolated from *A. terreus* [38].

In the same context, the mass ion peak at *m/z* 341.1782 [M−H]^−^ was identified as aspergillone **(16)** in match with the molecular formula C_21_H_26_O_4_, which was previously isolated from *A. versicolor* [39]. Another mass ion peak at *m/z* 341.1973 [M−H]^−^ was designed to be one of the two *γ*-butenolide derivatives, either spiculisporic acid C **(17a)** or D **(17b)**, which were previously reported from the urchin associated fungus *Aspergillus* sp. HDf2 and exhibited antibacterial activity against *S. aureus* ATCC 51650 [40]. The diketopiperazine alkaloid, protuboxepin G **(18),** was identified depending on the mass ion peak at *m/z* 374.1471 [M−H]^−^ and the molecular formula C_22_H_21_N_3_O_3_, that was characterized from the marine sponge-associated fungus *A. versicolor* SCSIO 41016 [41]. Similarly, the isoquinoline alkaloids, puniceusine E **(19)** and puniceusine J **(21)**, were annotated according to the mass ion peaks at *m/z* 377.1501[M+H]^+^ and 382.1251 [M−H]^−^, respectively, and in match with the molecular formulas C_22_H_20_N_2_O_4_ and C_21_H_21_NO_6_, respectively. Puniceusine E and J were reported from the fungus *A. puniceus* SCSIO z021 [30].

Moreover, the mass ion peak at *m/z* 382.2142 [M+H]^+^ was identified as tryprostatin A **(20)**, which was in agreement with the molecular formula C_22_H_27_N_3_O_3_ and was previously isolated from *A. fumigatus* [42]. Another mass ion peak at 389.3047 [M+H]^+^ was dereplicated as (5*S*,6*S*)-16,17-dihydroophiobolin H **(22)** according to the molecular formula C_25_H_40_O_3_. It was previously isolated form the fungus *A. insuetus* SD-512 and displayed broad-spectrum antibacterial activities against several bacterial strains, such as *Escherichia coli* QDIO-1, *Pseudomonas aeruginosa* QDIO-2 and many aquatic pathogenic micro-organisms [43]. Furthermore, the mass ion peak at *m/z* 433.2806 [M+H]^+^ was characterized as sclerotiotide E **(23)** according to the molecular formula C_23_H_36_N_4_O_4_ and was previously isolated from *A. sclerotiorum* PT06-1 [44]. Similarly, the prenylated indole alkaloid, 6-*epi*-avrainvillamide **(24)**, was identified depending on the mass ion peak at *m/z* 444.1955 [M−H]^−^ and the molecular formula C_26_H_27_N_3_O_4_. Compound **(24)** was previously reported from *A. taichungensis* [45]. Finally, the mass ion peak at *m/z* 472.346 [M−H]^−^was characterized as fiscpropionate D **(25)** in agreement with the molecular formula C_29_H_47_NO_4_. Fiscpropionate D **(25)** was isolated from *A. fischeri* FS452 and displayed a significant inhibitory effect against *Mycobacterium tuberculosis* [46].

### Anti- MRSA activity

The antibacterial potential of the extracted fungus was assessed using the pathogenic strain MRSA (ATCC 33,591), utilizing the IC_50_ of the quantitative antibacterial assay. The results of our study indicated that the fungal extract exhibited potent anti-MRSA efficacy with an IC_50_ value of 3.5 µg/mL in comparison with the IC_50_ value of ciprofloxacin (25.7 µg/mL). This indicated that the tested extract contained bioactive anti-MRSA metabolites.

### In silico studies

#### Construction of protein–protein interaction network

**Fig. 2:**
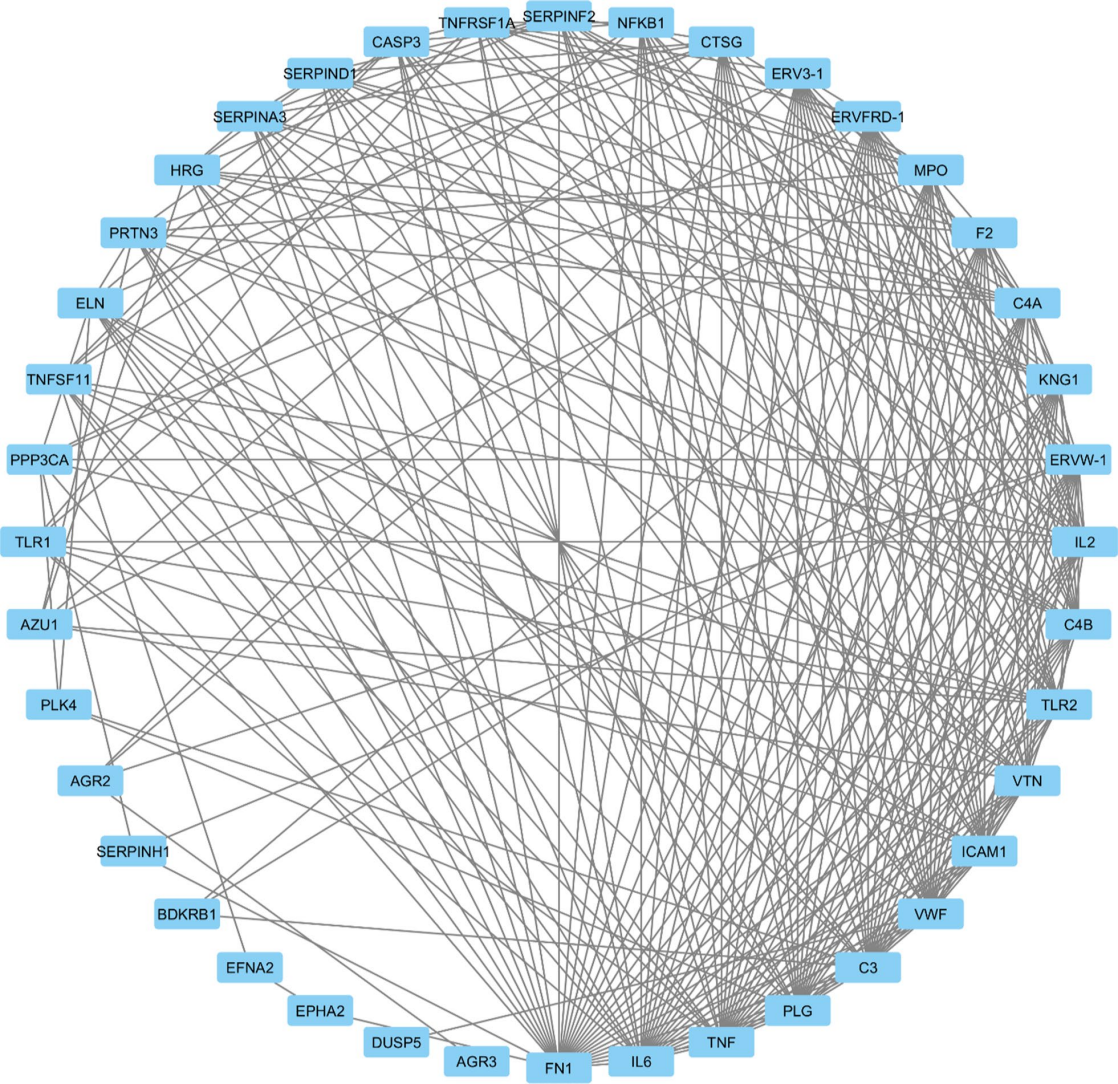
PPI network depicting the molecular mechanisms of antimicrobial action against MRSA

Proteins of interest were input into STRING database version 12.0 (accessible at https://string-db.org) to construct a foundational Protein–Protein Interaction (PPI) network, revealing their direct and functional linkages. Subsequent visualization of the PPI network was done using Cytoscape software version 3.10.1. The Cytoscape Analyzer was employed to craft a protein interaction map comprising 40 nodes and 277 edges, with an average node degree of 13.8. PPI network illustrates a highly interconnected system of molecular interactions, with a dense central region indicating the presence of key hub proteins that play critical roles in the underlying biological processes that are integral to combating MRSA infections.These Proteins such as **TNF**, **IL6**, and **FN1** are likely central regulators due to their position and high degree of connectivity, suggesting their involvement in critical pathways such as inflammation, immune response, or extracellular matrix regulation (Fig. 2).

#### Gene ontology enrichment analysis

**Fig. 3:**
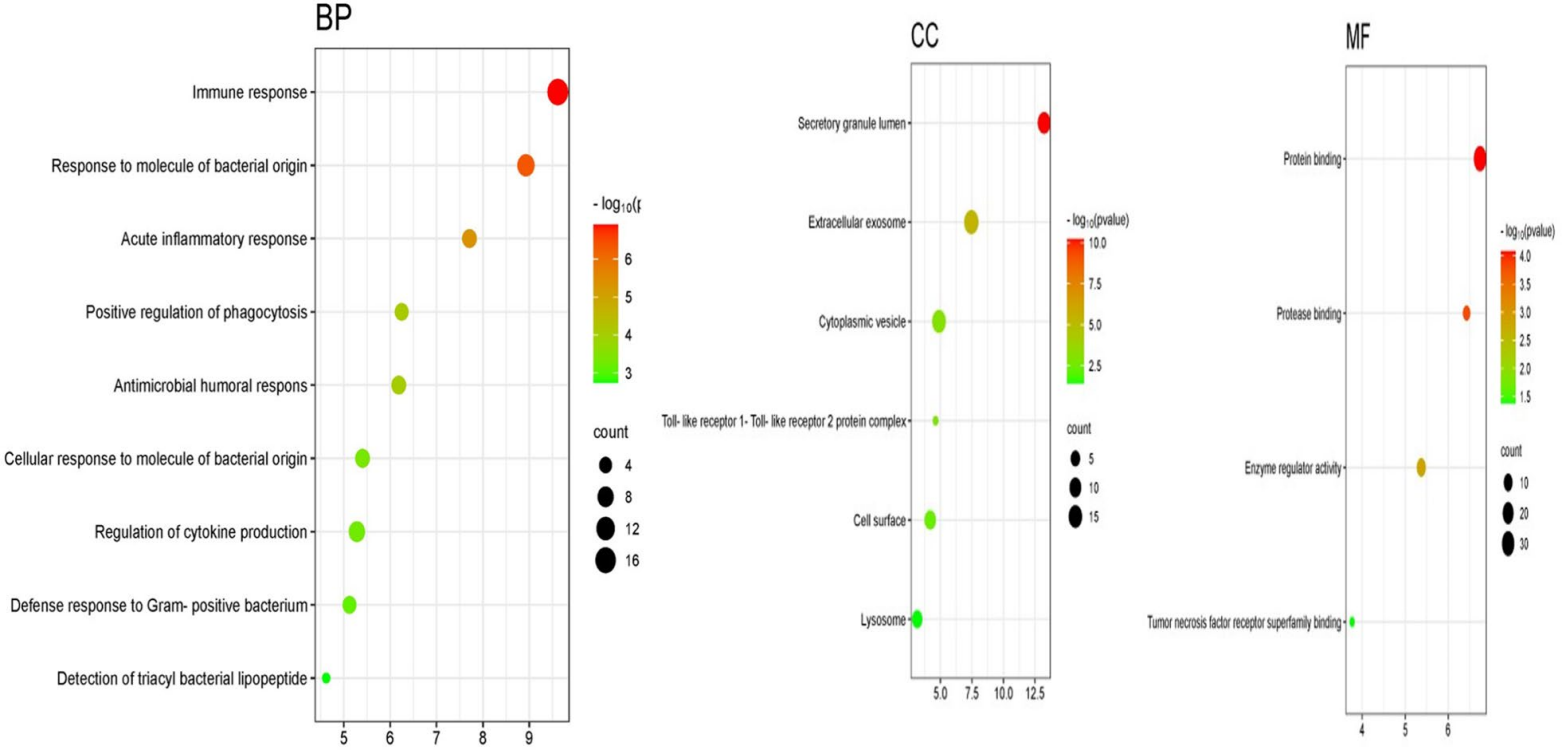
Bubble plot of gene ontology terms: biological process, cellular component, and molecular function

Through Shiny GO v0.80, an enrichment analysis of GO was conducted to elucidate the biological attributes of overlapping proteins in our dataset. This thorough exploration unveiled 334 BP, 23 CC, and 16 MF terms, all were statistically significant with *p*-values under 0.05, and systematically documented in supplementary (Table S1). This analysis outlines a triad of responses integral to combating MRSA infections. The implicated proteins are crucial for both the recognition of bacterial antigens and the subsequent activation of the immune defenses, highlighting an intricate immune defense system. The initiation of immune responses is set off by the recognition of bacterial entities such as triacyl lipopeptides, leading to the activation of cytokine production and phagocytosis pathways that are key for managing and resolving the infection. Notably, the activity of TNF receptor binding within the MF terms is of particular significance due to its role in inflammation and apoptosis, which are essential for resolving bacterial infections, MRSA included. The BP terms emphasize the importance of the antimicrobial humoral response as a vital defense, particularly against antibiotic-resistant MRSA strains, involving antibody production and the activation of complement pathways to neutralize and eradicate the bacteria. The CC terms point to lysosomes and secretory granules as critical sites within the cellular framework for antigen processing and presentation, as well as for the degradation of engulfed bacteria (Fig. 3). These GO terms provide insights into potential drug targets. Enhancing protease binding activities could lead to new treatments that prevent MRSA from circumventing immune detection. Targeting the TLR protein complex could increase immune system recognition of MRSA, potentially countering its pathogen resistance. Additionally, the GO terms associated with cytokine production regulation and bacterial response hint at strategies to manage MRSA-induced inflammation, which can cause significant tissue damage. Regulating cytokine levels may help balance the immune response to effectively address the MRSA challenge, while minimizing collateral damage. In summary, the GO enrichment analysis offers an essential framework for decoding the complex biological dynamics involved in MRSA infections. It highlights the potential for developing targeted therapies that bolster the immune response or reduce inflammation, offering hope for more effective treatment against this challenging pathogen.

#### Analysis of enriched KEGG pathways

**Fig. 4:**
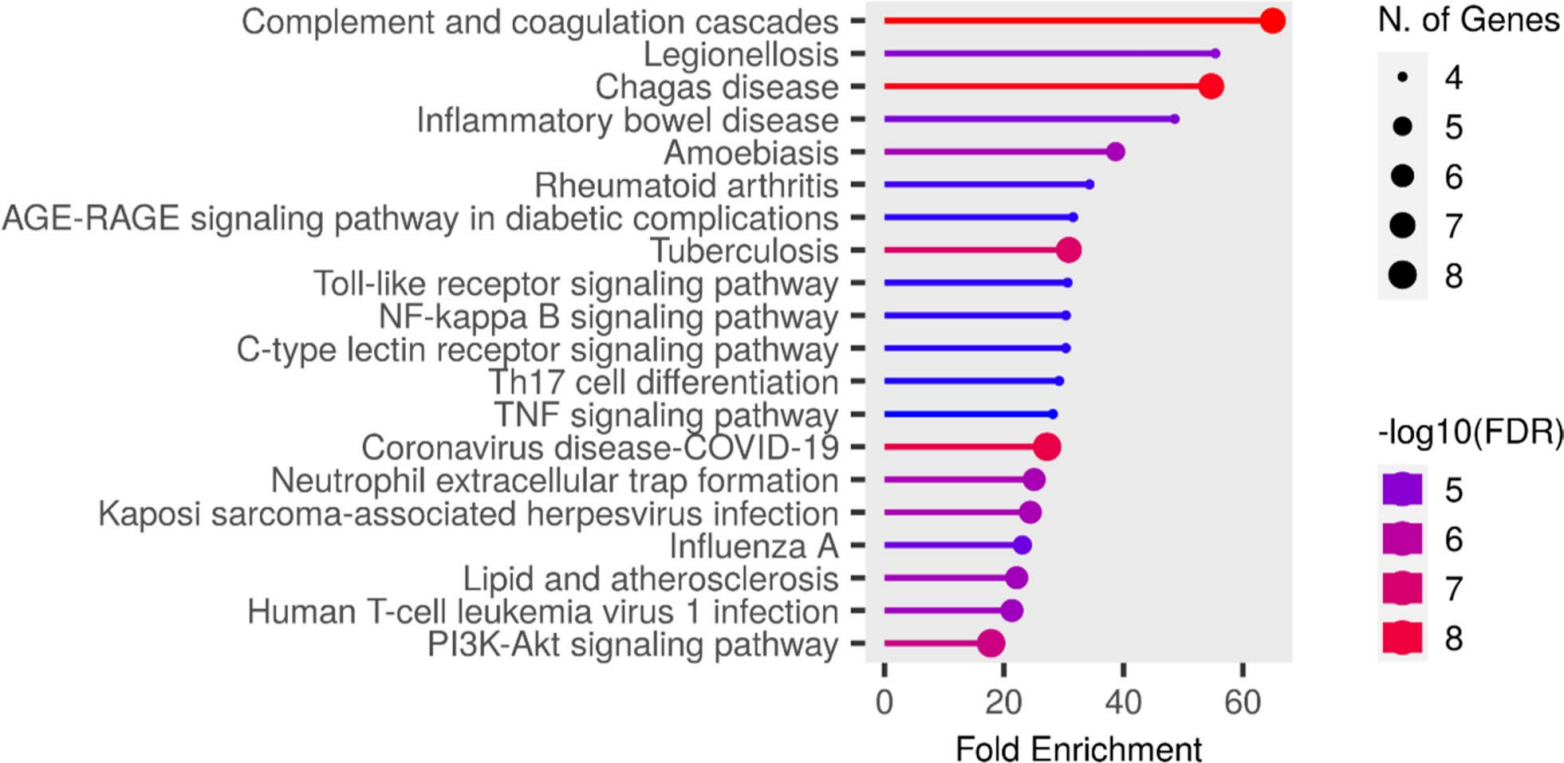
KEGG barplot of our network genes

The KEGG enrichment analysis plays a key role in linking protein targets to distinct molecular pathways. Within our study, this method has illuminated the pathways potentially engaged by protein targets pertinent to the chosen bioactive compounds in relation to MRSA infection, as detailed in Table S2. The outcomes of the KEGG pathway enrichment analysis are visually presented in a bar plot, denoted as (Fig. 4). This analysis is essential for identifying the biological pathways that are significantly influenced by a specific group of genes. Notably, the TLR signaling and TNF signaling pathways are suspected to be involved in the pathogenesis of MRSA.

**Fig. 5:**
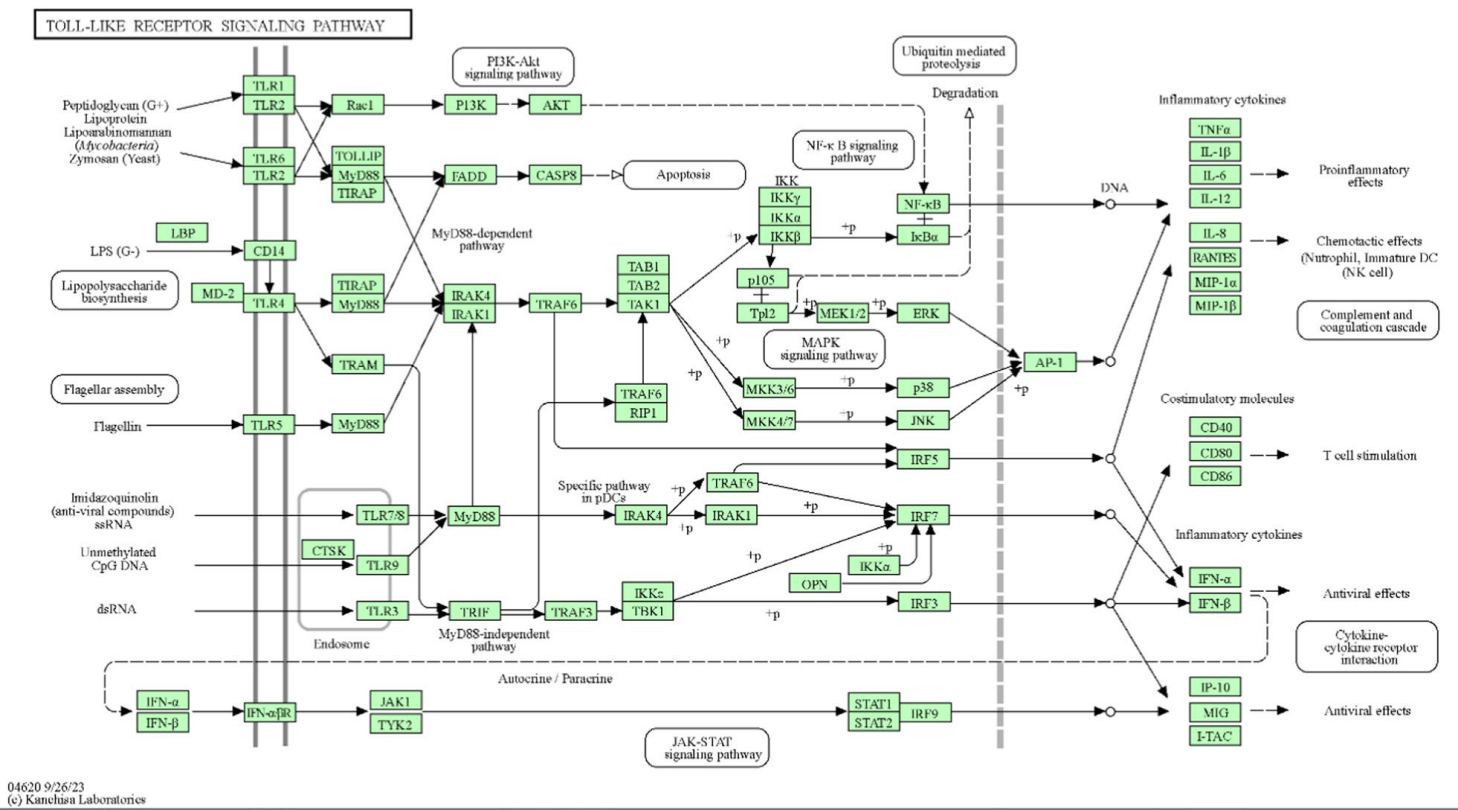
Modulation of TLR pathway against MRSA

MRSA triggers immune defenses through TLR2 recognition of its components, like lipoteichoic acid and peptidoglycan. This sets off a chain reaction, where proteins like MyD88 activates other molecules that switch on critical pathways, including NF-κB and MAPKs, leading to the release of inflammatory substances that help fight the infection (Fig. 5). However, when this response is too strong, it can harm the body, causing inflammation that results in tissue damage. Finding agents that can keep this immune response in check could offer a promising way to deal with MRSA, especially since it often doesn't respond to classical antibiotics. Targeting the immune response pathways, such as enhancing phagocytosis or modulating cytokine responses, presents an alternative strategy to combat these infections.

**Fig. 6:**
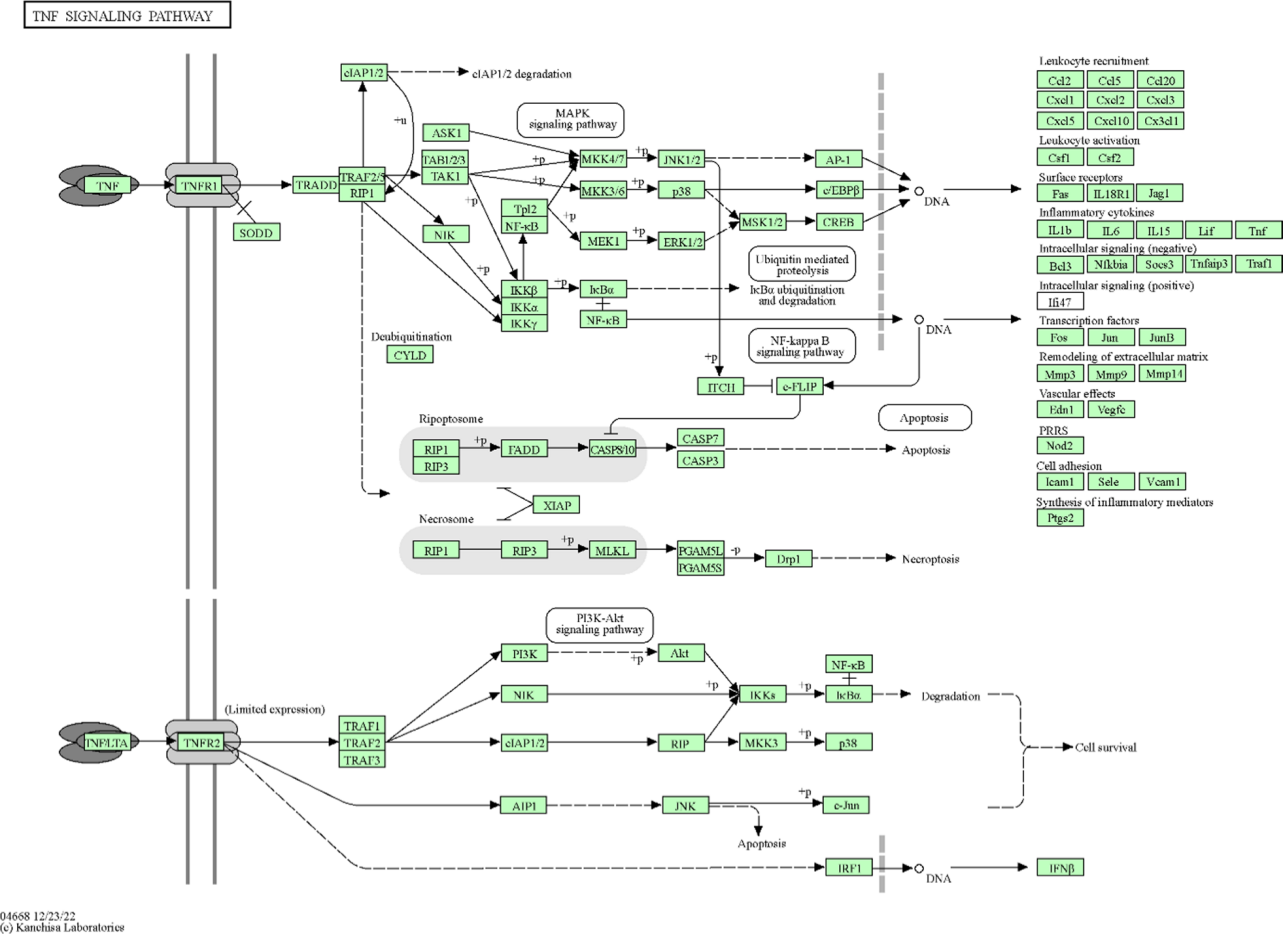
Modulation of TNF signaling pathway against MRSA

The TNF signaling pathway plays a pivotal role in the host response to MRSA infections. Initiation of this pathway occurs when TNF binds to the TNFR1 receptor, which then recruits adaptor proteins such as TRADD and TRAF2. This recruitment cascade triggers further downstream signaling events, particularly the activation of the NF-κB pathway, a crucial regulator of genes involved in the immune response. As a result, a variety of inflammatory cytokines are produced (Fig. 6). Simultaneously, TNF signaling can induce apoptosis, serving as a double-edged sword that can both restrict infection spread by removing infected cells and contribute to tissue damage if uncontrolled. The impact of TNF signaling on MRSA infections is thus complex; it can effectively mobilize leukocytes to the infection site, aiding in bacterial clearance, while its overactivation can cause rampant inflammation and consequent tissue damage, which are hallmarks of severe MRSA infections. Modulating the TNF pathway represents a therapeutic opportunity in MRSA treatment strategies. Pharmacological agents,designed to temper the TNF response, could reduce the inflammatory damage associated with MRSA infections without compromising the immune capacity to eliminate the bacteria. Therefore, understanding and controlling the dynamics of TNF signaling is crucial for developing interventions that can both manage inflammation and enhance bacterial clearance in MRSA infections.

#### Identification of central hub genes

In our PPI network, comprised of 40 nodes and 276 edges, we focused on identifying key hub genes, which are nodes with extensive connections to other proteins, signifying their critical roles in the network. Using the CytoHubba plugin in Cytoscape, we applied several ranking algorithms—degree, edge percolated component (EPC), maximum neighbourhood component (MNC), and maximal clique centrality (MCC), along with closeness, radiality, betweenness, and stress—to determine the top 10 hub genes based on their network connectivity.

Our analysis revealed that certain nodes were consistently identified as hubs across multiple analytical methods, underscoring their significance (Table 2).

**Table 2:** Recurrence count of protein-coding genes identified by eight different methods in the CytoHubba Plugin for Cytoscape

No	Name	Occurrence
1	TNF	8
2	TLR2	8
3	VWF	8
4	C3	8
5	IL-6	7
6	FN1	7
7	ERVW-1	7
8	PLG	6
9	VTN	6
10	1CAM1	5

**Fig. 7:**
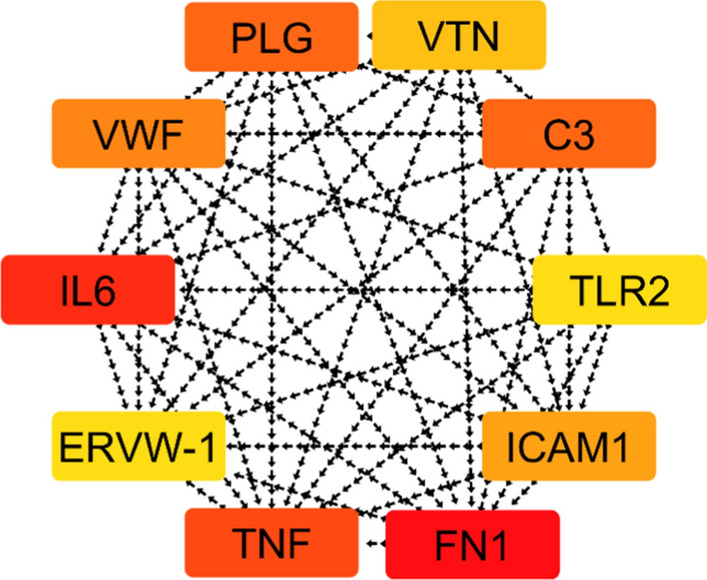
Top 10 Hub Genes: color intensity indicates strength of connectivity

Specifically, TLR2, TNF, VWF, and C3 were recognized as the most prominent hub genes, each appearing in all eight ranking techniques employed. Following closely were IL6, FN1, and ERVW-1, each scoring seven times across the methods, indicating their substantial connectivity within the PPI network. PLG and VTN were identified six times, and ICAM1 was highlighted five times, as shown in the supplementary information (Figures S5 and S6). This systematic approach to network analysis underscores the importance of these genes in the PPI network and potentially, in the biological processes related to their functions. TLR2, VWF, and TNF emerged as the most prominent hub genes within the network analysis (Fig. 7). TLR2 is a crucial component of the innate immune system, detecting MRSA cell wall components and triggering an immune response. VWF is critical for MRSA adhesion, platelet aggregation, and thrombus formation. TNF, a pro-inflammatory cytokine, is vital for bacterial infection control but may also intensify inflammation, contributing to MRSA-induced pathology. As a result, they were subsequently selected for in-depth, in silico molecular modeling. This selection is based on their critical number of interactions within the network, which suggests they may play significant roles in the biological processes of MRSA infection.

#### Molecular modeling studies

Molecular modeling of pivotal proteins such as TLR2, VWF, and TNF offers an advanced avenue for understanding the intricacies of immune response and coagulation processes in the presence of MRSA infections. By simulating the molecular structures and affinities of these hub genes, we can predict their interactions and functions with high precision. Integrating this with the docking studies of PBP2a (a key factor in the antibiotic resistance of MRSA) provides a complementary in silico approach that marries detailed protein network analysis with targeted drug design. This dual methodological strategy holds the potential to uncover novel therapeutic avenues, addressing the challenge of MRSA by targeting both the pathogen resistance and the host immune response. Consequently, modeling studies were conducted on the binding sites of TLR2, VWF, TNF, and PBP2a to evaluate the binding affinity of 27 metabolites, identified from *Aspergillus* sp*.* extract.

#### Molecular modeling with TLR2

**Fig. 8:**
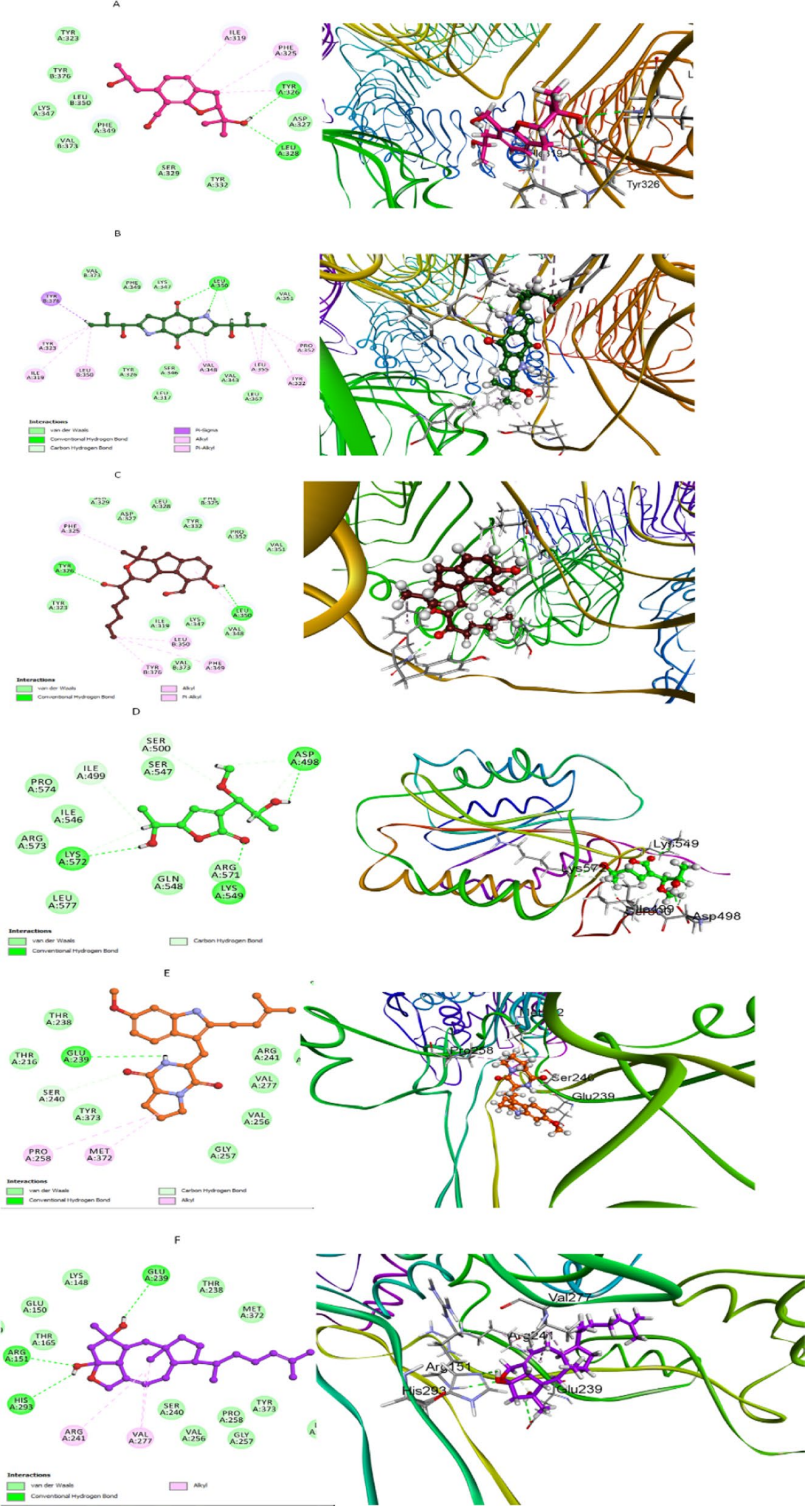
2D and 3D docking of compounds **9** (**A**), **15** (**B**) and **16** (**C**) within the binding pocket of TLR2 (PDB ID: 6NIG). **D** Compound **2** docking in the active site of VWF (PDB ID: 1AUQ). Compounds **20** (**E**) and **22** (**F**) docking within the active pocket of PBP-2a (PDB ID: 1VQQ)

The molecular docking approach was used to evaluate the potential interactions between the identified metabolites and TLR2 enzyme. Redocking of the ligand into the TLR2 active site yielded RMSD of 1.635 Å and a binding energy of −5.532 kcal/mol. As observed in Table S3, all the listed compounds showed moderate to promising binding affinities to TLR2 with comparable, or even lower, binding energies (−5.108 to −9.747 kcal/mol) to that of the corresponding ligand (−5.532 kcal/mol). In selecting a compound from Table S3 for further investigation, JBIR-75 (compound 5) was chosen based on its promising binding affinities, RMSD values, and molecular weight when interacting with the TLR2 enzyme. This compound exhibited a moderate binding energy of −5.392 kcal/mol against TLR2, which is close to the binding energy of the control ligand (−5.532 kcal/mol). Notably, the RMSD value for JBIR-75 is 1.420 Å, suggesting a stable and reliable docking position compared to other compounds with higher RMSD values. The Root Mean Square Deviation of a docked ligand is an essential metric that measures the average distance between the atoms of the ligand in its docked conformation and its reference structure. A lower RMSD value generally indicates a more accurate and successful prediction of the conformation within the binding site after docking. Furthermore, the molecular weight of JBIR-75 is another critical factor to consider, as it influences the pharmacokinetic properties of the compound. The optimal molecular weight enhances the likelihood of the compound being a successful therapeutic agent due to better cell membrane permeability and reduced metabolic alterations. Additionally, Terreusinone **(15)** and Aspergillone **(16)** were also considered due to their competitive binding energies of −6.799 and −6.364 kcal/mol, respectively, against TLR2. These values are significantly better than that of the control ligand. The RMSD values for Terreusinone and Aspergillone are 1.267 and 0.885 Å, respectively, indicating highly accurate docking conformations. These compounds also maintain advantageous molecular weights, which might contribute to favorable pharmacokinetic profiles, enhancing their potential as therapeutic agents. These compounds formed multiple bonding interactions with key residues TYR 326, LEU 328, LEU 350, PHE 325, VAL 348, and ILE 319, as illustrated in (Fig. 8).

#### Molecular modeling with VWF

The data presented in Table S3 indicated that most of the docked metabolites displayed moderate to promising VWF inhibitory potential compared to the corresponding reference ligand (S = −6.75 kcal/mol), with binding energy scores in the range of −3.97 to −6.827 kcal/mol, except for aspilactonol D (**2**) that demonstrated the highest binding ability within the active site of TNF-a (S = −7.539 kcal/mol), showing different interactions with the amino acid residues ASP 498, ARG 571, LYS 572, and SER 500, which might suggest its ability to inhibit MRSA caused by excessive VWF release (Fig. 8). Additionally, Aspilactonol D **(2)** exhibited a RMSD value of 1.056 Å in its docking with TNF-α, indicating a stable and accurate binding conformation relative to its reference structure.

#### Molecular modeling with TNF-α

The results of the current docking approach indicated the possible binding affinity of fiscpropionate D (**25**) to the active site of TNF-α, showing a lower energy score (S = −6.198 kcal/mol) than that of the co-crystallized ligand (S = −5.963 kcal/mol), followed by sclerotiotide E (**23**) and puniceusine J (**21**) (S = −**5.932**and −**5.723** kcal/mol, respectively), suggesting their possible impact on the antibacterial potential via targeting TNF-α enzyme (Table S3). The RMSD values provide further insight into the stability and accuracy of these interactions. fiscpropionate D (**25**) demonstrated a RMSD of 1.147 Å, which indicates a highly reliable docking position relative to the native structure of the ligand in TNF-α. Similarly, sclerotiotide E (**23**) and puniceusine J (**21**) showed RMSD values of 1.144 and 1.886 Å, respectively.

#### Molecular modeling with PBP-2a

Docking studies of the antibiotic Ampicillin into the active site of PBP-2a yielded a RMSD of 1.659 Å and a binding energy of −6.232 kcal/mol, confirming the reliability of the docking procedure. A series of twenty-seven compounds were subsequently screened for binding to PBP-2a, with results tabulated in Supplementary (Table S3). Tryprostatin A **(20)** and (5S,6S)-16,17-dihydroophiobolin H **(22)** exhibited docking scores of −7.02885 and −6.85122 kcal/mol, respectively, surpassing the binding affinity of ampicillin. The interaction profiles of these compounds with PBP-2a were characterized by multiple bonding interactions with critical active site residues, specifically GLU 239, ARG 151, HIS 293, MET 372, PRO 258, and VAL 277. These interactions, which are key determinants of binding affinity, were graphically represented in Fig. 8. Tryprostatin A **(20)** displayed a RMSD of 1.5908194 Å, and 5S,6S)-16,17-dihydroophiobolin H **(22)** had a RMSD of 1.7428789 Å. These RMSD values, though slightly higher than that of Ampicillin, still indicate well-fitted conformations within the active site of PBP-2a, enhancing our confidence in their potential efficacy (Fig. 8). The enhanced binding affinities of these compounds suggest their potential as lead molecules for the development of new antibacterial agents targeting MRSA.

## Materials and methods

### Plant material

Healthy fresh *A. graveolens* seeds were obtained from Minia area, Egypt in September 2022 and were identified by Prof. Dr. Nasser Barakat (Professor of Botany, Faculty of Science, Minia University).

### Endophytic fungus strain isolation and purification

Following previously reported procedures of isolation [47, 48], the endophytic fungal strains were isolated from the tissues of inner roots under sterile conditions with some modifications as follows; the fresh plant roots were collected, washed with tap water for 5 min, and then washed with sterilized distilled water for another 5 min. After that, the roots were soaked in ethanol 70% for 2 min, and finally washed with distilled sterilized water. In the next step, the roots were dissected with a sterile scalpel under aseptic conditions and surface placed on Potato Dextrose Agar medium (PDA, 200-g potato extract, 20 g glucose, and 15 g agar–agar powder in 1 L distilled water, PH 6.0) supplemented with Gentamycin (100 mg/L) and amoxicillin (100 mg/L) to overcome any bacterial growth and incubated at 30 °C for up to two weeks and observed frequently for any growth. Each pure fungal colony was isolated, and surface streaked again in order to obtain pure fungal isolates with certain morphological characteristics. The purified strains were kept as a glycerol stock at −70 °C.

### Molecular identification and phylogenetic analysis

The fungal strain SH1 was identified genetically using sequence analysis of the internal transcribed spacer (ITS) region, which includes ITS1, 5.8S rRNA gene, and ITS2 sequences, as well as the partial 18S rRNA gene, according to [49, 50]. Briefly stated, the MasterPure Yeast DNA extraction kit (Epientre, Madison, Wisconsin) was used to extract DNA from fungal biomass. Using the universal fungal primers NS1 [51] and ITS-4 [52], DNA amplification of the 18S rRNA gene and the complete ITS region was carried out. Sanger sequencing was performed with primers ITS-4 (ITS sequence analysis) and NS1 (partial 18S rRNA gene sequence analysis) by LGC Genomics (Berlin, Germany). Manual sequence corrections and phylogenetic analysis were performed with MEGA11 version 11.0.1 [53, 54]. The RefSeq Targeted Loci project databases (BioProjects PRJNA39195 and PRJNA177353; both updated on May 13, 2024) were used to identify next-related strains using the BLASTn tool from the NCBI (https://www.ncbi.nlm.nih.gov/). The next-type material strains' ITS and 18S rRNA gene sequences were loaded into MEGA11 and aligned using ClustalW [55]. Uniform rates were applied at all nucleotide sites, and paired deletions were used to compare sequences. The Kimura 2-parameter model [56] for the ITS sequence and the General Time Reversible model [57] for the 18S rRNA gene were used in the construction of the phylogenetic trees, along with the maximum likelihood technique. The bootstrap technique (100 replications) was used to test the phylogenies. For the examination of the ITS and 18S rRNA gene sequence-based analysis, a total of 50 sequences with 562 nucleotide positions and 43 sequences with 1158 nucleotide positions were taken into consideration. Both sequences obtained for the new fungal strain were submitted to GenBank/EMBL/DDBJ under the accession numbers PP790407 (ITS sequence) and PP790381 (18S rRNA gene sequence).

### Fungal strain fermentation and extract preparation

Large-scale fermentation was performed on solid rice medium (prepared by adding 100 mL of distilled water to 100-g rice in a 1-L Erlenmeyer flask and macerated overnight before autoclaving) as follows: The pure identified fungal strain in this study was cultured on PDA agar and incubated till healthy growth was obtained, then a ratio of 1.5 plate of the strain was added to every 1 sterilized rice flask and kept for 30 days at room temperature. After that, the fermentation of the fungus on the rice was stopped by adding ethyl acetate in a quantity sufficient to immerse the fermented rice. In the next step, the rice was crushed into small pieces as possible, and the extraction continued three times in three days till complete exhaustion. The ethyl acetate extract was then filtered out and concentrated by a rotary evaporator.

### Metabolomic analysis

Mass spectrometry analysis was performed by dissolving the fungal strain's ethyl acetate extract in 1 mg/mL of methanol. Then, the metabolomic analysis was performed using LC-HR-ESI-MS in compliance with the previously mentioned methodology [58]. The Acquity Ultra Performance Liquid Chromatography system and Synapt G2 HDMS quadrupole time-of-flight hybrid mass spectrometer (Waters, Milford, MA, USA) were used for the LC-HRMS analysis. The BEH C18 column was connected to the guard column and set to 40 °C so that the sample (2 µL) could be injected. Both positive and negative ionization modes were used, along with a spray voltage of 4.5 kV. The capillary temperature was adjusted to 320 °C and the mass range to m/z 150–1500. After processing the MS dataset, data were extracted using MZmine 2.20 according to the established parameters. Molecular formula prediction and peak identification were then applied to the processed data set. The DNP (Dictionary of Natural Products) and MarinLit were used to annotate the identified constituents.

### Anti- MRSA activity

The antibacterial activity of *Aspergillus* sp. extract was evaluated against MRSA (ATCC 33,591) using microtiter bioassay [59]. The dried fungal extract was dissolved in dimethyl sulfoxide (DMSO) to a concentration of 100 mg/mL. The bacterial strain was adjusted to 1 McFarland standard, which is equivalent to 3.0 × 10^8^ CFU/mL, using overnight culture in Mueller Hinton broth (Sigma Aldrich, SA) at 37 °C in a shaker incubator. Initially, 100 µL of sterile Mueller Hinton broth was added to each well on a microtiter plate. One hundred microliters (100 µL) of the examined extract was introduced into the first well, and from there, the extract was serially diluted by 100 µL (1:1) through the 8th well. The last well’s 100 µL was discarded. Consequently, 50, 25, 12.5, 6.25, 3.125, 1.5625, 0.78125, and finally 0.3906 mg/mL were the concentrations of the tested extract. Each well of a microtiter plate received 5 µL of the bacterial solution, except for the raw of sterility control. Using the same concentrations as the examined extract, the antibiotic ciprofloxacin was utilized as a positive control for the bacterial strain [60]. Raw simply contains sterile, simple medium, and a microorganism was implemented as a negative control. The microtiter plates were incubated for 24 h at 37 °C. Following incubation, an ELISA plate reader was used to measure the plate at a wavelength of 570 nm.

### In silico* study*

#### Developing a protein–protein interaction network

To explore the molecular interactions relevant to MRSA treatment, common genes identified through research were input into the STRING database [61], a well-known resource for assembling protein–protein interaction (PPI) networks. MRSA, a notable pathogen due to its drug resistance, was the focal point of the search within the human host context. PPI are of high value due to their specificity and versatility, playing an essential role in understanding the biological mechanisms underlying MRSA resistance. A stringent interaction score threshold was applied to ensure the reliability of the PPI network, with only interactions above a 0.4 combined score being considered. Subsequent network visualization and analysis were conducted using Cytoscape software, which is recognized for its efficacy in network analysis [62]. The analysis aimed to identify hub genes—key proteins with numerous connections to others in the network. These hubs often have critical functions and are central to the network's integrity, reflecting their importance in biological processes. By utilizing the CytoHubba/Cytoscape plugin, we focused on degree-based methods to discern the pivotal components within the PPI network. This approach shed light on the primary genes involved in MRSA network, enhancing our understanding of potential targets for more effective MRSA treatments. These hub genes may offer insights into new therapeutic avenues, potentially leading to breakthroughs in combating MRSA infections.

#### Examination of gene ontology (GO) and Kyoto Encyclopedia of genes and genomes (KEGG) pathway enrichment

To investigate the functions and interactions of genes pertinent to MRSA, Gene Ontology (GO) and pathway enrichment analyses were performed. These analyses provided a threefold perspective: Biological Processes (BP) described the roles of genes in various biological activities; Cellular Components (CC) pinpointed the exact locations within the cell, where genes or proteins are active; and Molecular Functions (MF) characterized the specific activities and interactions at the molecular level. Utilizing Shiny GO (http://bioinformatics.sdstate.edu/go/), a web-based tool for GO analysis with a stringent False Discovery Rate (FDR) cutoff of less than 0.05, was allowed for the identification of statistically significant attributes and pathways. The results were then illustrated through enrichment bubble plots using SR plot (https://www.bioinformatics.com.cn/en), an online visualization resource. This approach facilitated a comprehensive understanding of the roles and interactions of genes implicated in MRSA, enabling the identification of potential targets for therapeutic intervention.

#### Molecular docking studies

Molecular docking simulation was performed to predict and evaluate the binding abilities of the characterized metabolites towards toll-like receptor (TLR2; PDB ID: 6NIG), von willebrand factor (VWF; PDB ID: 1AUQ), tumor necrosis factor-alpha (TNF-α; PDB ID: 2AZ5), and penicillin binding protein 2a (PBP-2a; PDB ID: 1VQQ). The targeted metabolites were presented in a 2D model using the CHEMDRAW software, where the energies of the proposed structures were minimized to attain the most stable conformers that were kept in a database for docking studies. The X-ray crystallographic structures of the target enzymes were obtained from the RCSB Protein Data Bank (http://www.rcsb.org/). Validation of the structures of the prepared proteins was done via re-docking of the co-crystallized ligands and measuring both their docking score (S; kcal/mol) and root-mean square deviation value (RMSD; Å). A visual examination of docking poses with the lowest binding free energy (expressed as the docking score of each molecule) was chosen, and the best ones were utilized to produce interaction diagrams. Discovery Studio Client was the chosen platform for carrying out the molecular docking simulations [63]. This process validated our network analysis, pinpointing viable drug candidates for MRSA treatment and informing future drug combination strategies in accordance with previously published procedures [64].

## Conclusion

Our study unveils a promising avenue for discovering novel anti-MRSA drugs from fungal endophytes. Metabolomic profiling using HRESIMS identified a diverse range of 27 bioactive compounds in the *Aspergillus* sp. endophyte associated with *Apium graveolens* seeds. Notably, the crude extract exhibited potent anti-MRSA activity (IC_50_ = 3.5 µg/mL), significantly surpassing ciprofloxacin (IC_50_ = 25.7 µg/mL). Further in silico analysis using molecular docking provided valuable insights. The Protein-Interaction network revealed TNF, TLR2, VWF, and PBP2a as central hubs in the MRSA immune response. Interestingly, eight compounds (**2, 9, 15, 16, 20, 22, 23,** and **25**) from the *Aspergillus* sp. extract displayed enhanced binding affinities to these potential targets. These findings suggest their potential as lead molecules for developing new anti-MRSA therapeutics.

Future research should focus on isolating and purifying these promising bioactive compounds (compounds 2**, 9, 15, 16, 20, 22, 23,** and **25**) for further characterization and in vivo evaluation. Additionally, exploring the synergistic effects of these compounds with existing antibiotics could be a valuable strategy. Our study underscores the immense potential of fungal endophytes as a rich source of novel anti-MRSA agents, warranting further investigation in the fight against antibiotic resistance.

## Supplementary Information

Additional file 1.

## Data Availability

No datasets were generated or analysed during the current study.
