# Supplementary material for: Apium graveolens-associated Aspergillus sp.: metabolomic profiling and anti-MRSA potential supported by in silico studies
Source: Microb Cell Fact. 2025 Mar 8;24:57. doi: 10.1186/s12934-025-02645-9 (PMC11889860; doi:10.1186/s12934-025-02645-9)
Supplement: Supplementary file 1 — Additional file 1. [file 12934_2025_2645_MOESM1_ESM.docx]

**Supplementary information**

***Apium graveolens*-associated *Aspergillus* sp.:** **Metabolomic Profiling and Anti-MRSA Potential** **Supported by *In Silico* Studies**

Alshymaa Abdel-Rahman Gomaa^1^, Hesham A. Abou-Zied^2^, Sara Mahmoud Farhan^3^, Ruqaiah I. Bedaiwi^4^, Mohammad A. Alanazi^4^, Stefanie P. Glaeser^5^, Peter Kämpfer^5^, Usama Ramadan Abdelmohsen^1,6,^*, Fatma Alzahraa Mokhtar^7,8 ¥,^, Enas Reda Abdelaleem^1¥^

^1^ Department of Pharmacognosy, Faculty of Pharmacy, Minia University, Minia 61519, Egypt.

^2^ Department of Medicinal Chemistry, Faculty of Pharmacy, Deraya University, Minia 61111, Egypt.

^3^ Department of Microbiology and Immunology, Faculty of Pharmacy, Deraya University, Minia 61111, Egypt

^4^ Department of Medical Laboratory Technology, Faculty of Applied Medical Sciences, University of Tabuk, Tabuk 71491, Saudi Arabia

^5^ Institute of Applied Microbiology, Justus-Liebig University Gießen, Gießen, Germany

^6^ Deraya Center for Scientific Research, Deraya University, Minia, 61111, Egypt

^7^ Department of Pharmacognosy, Faculty of Pharmacy, El Saleheya El Gadida University, El Saleheya El Gadida 44813, Sharkia, Egypt

^8^ Fujairah Rsearch Centre, Sakamkam Road, Sakamkam, Fujairah 00000, UAE

***** Correspondence: [usama.ramadan@mu.edu.eg](mailto:usama.ramadan@mu.edu.eg) (U.R.A.)

^¥^ both authors are equally contributed

**Figure S1:** Phylogenetic placement of fungal strain SH1 based on ribosomal ITS1 and ITS2 sequences located at the ribosomal rRNA operon between the 18S and 28S rRNA genes. The phylogenetic tree was calculated in MEGA 11 (Tamura et al. 2021; Stecher et al 2020) using the Maximum-Likelihood method and the Kimura 2-parameter model [[1](#_ENREF_1)]. Reference sequences were obtained from the Fungal Internal Transcribed Spacer RNA (ITS) RefSeq Targeted Loci Project (PRJNA177353). This analysis involved 50 nucleotide sequences. There were a total of 562 positions in the final dataset. Numbers at nodes represent bootstrap values (100 replications) of 70% and above. Numbers in brackets represent Acc. numbers of respective sequences. Bar: 0.01 number of nucleotide substitution per nucleotide side.

**Figure S2:** Phylogenetic placement of fungal strain SH1 based on partial sequences of the 18S rRNA gene. The phylogenetic tree was calculated in MEGA 11 ([[2](#_ENREF_2)]; [[3](#_ENREF_3)]) using the Maximum-Likelihood method and the General Time Reversible model [[4](#_ENREF_4)]. Reference sequences were obtained by NCBI BLAST analysis against the reference sequences of the Fungal 18S Ribosomal RNA (SSU) RefSeq Targeted Loci Project database (PRJNA39195). Only a reduced number of *Aspergillus* type material sequences was in the database. This analysis involved 43 nucleotide sequences. There were a total of 1158 positions in the final dataset. Numbers at nodes represent bootstrap values (100 replications) of 70% and above. Numbers in brackets represent Acc. numbers of respective sequences. Bar: 0.01 number of nucleotide substitution per nucleotide side.


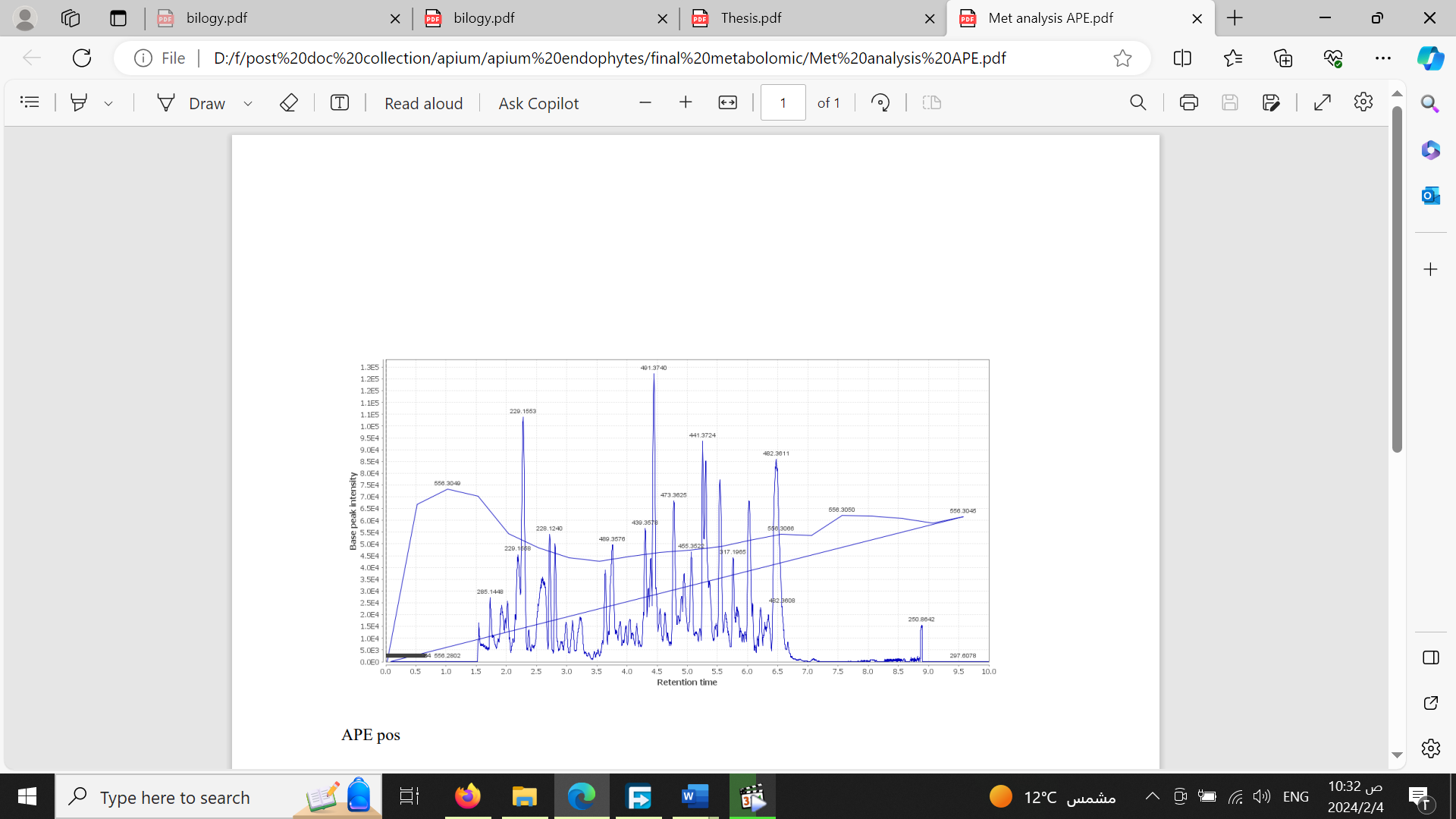


**Figure S3**: Total ion chromatogram for the extract of *Asperillus* sp. on positive ionization mode.


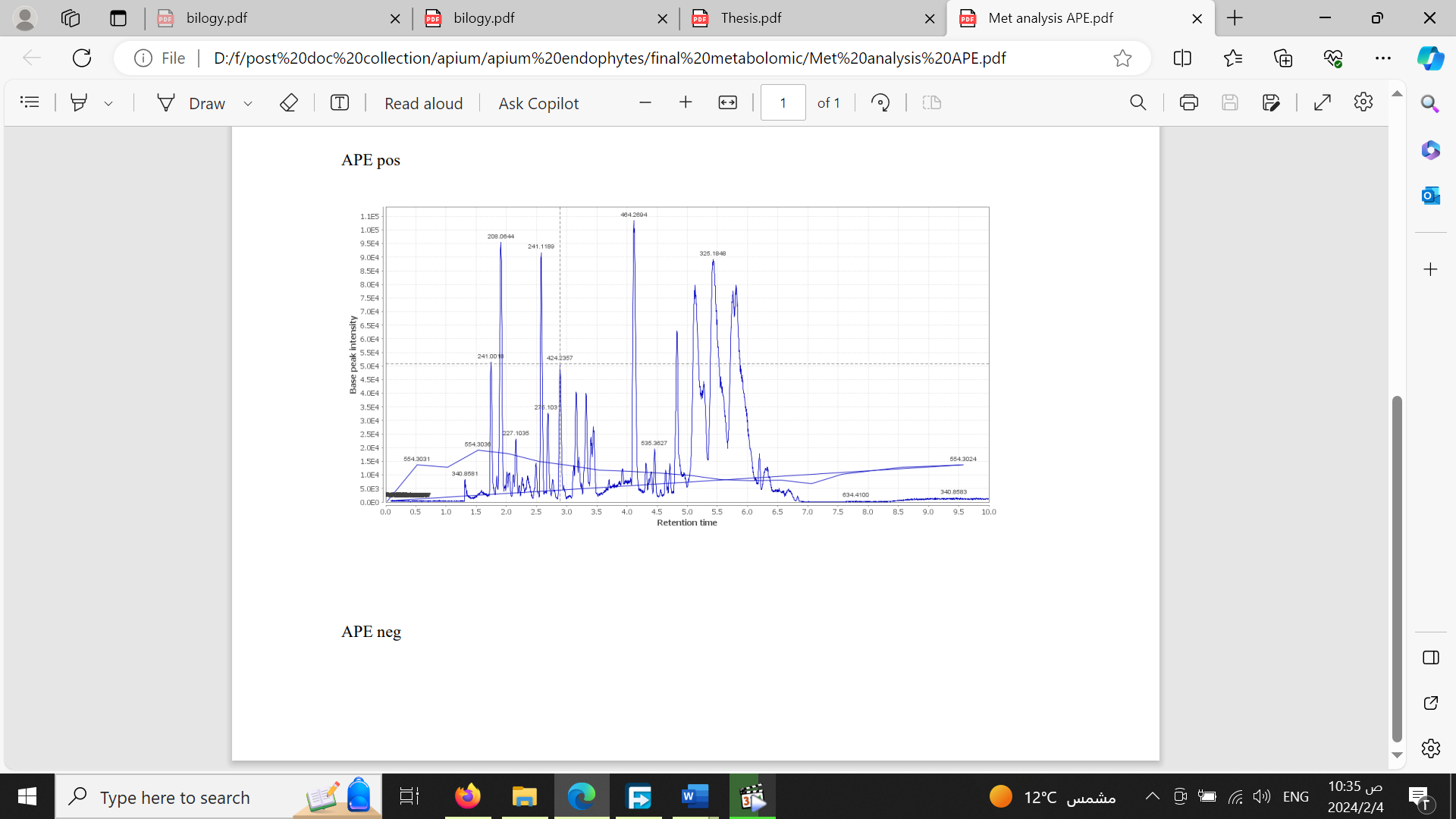


**Figure S4**: Total ion chromatogram for the extract of *Asperillus* sp. on negative ionization mode.


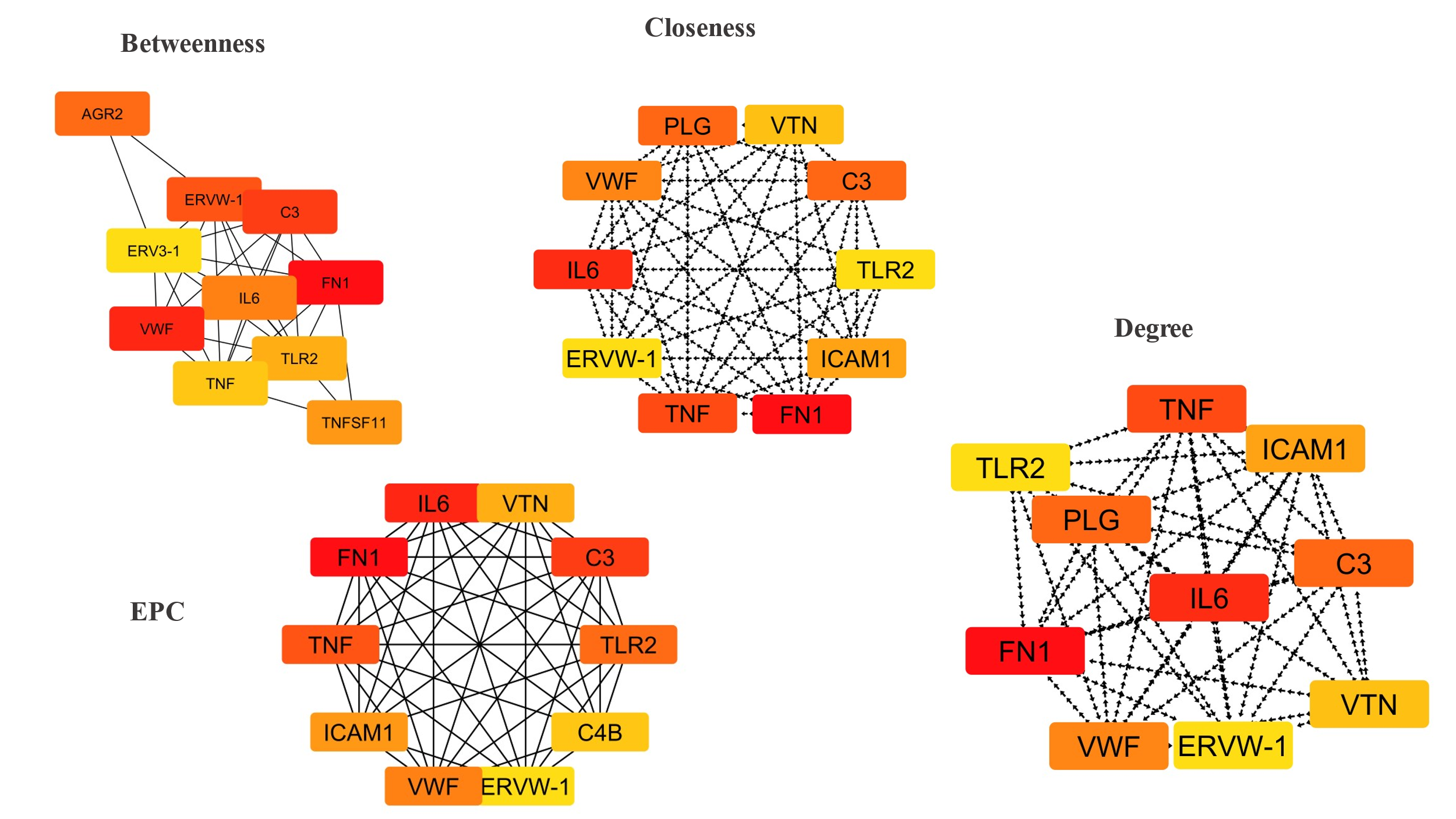


**Figure S5:** The occurrence of 10 hub genes by different 4 methods in analysis of cytoHubba.


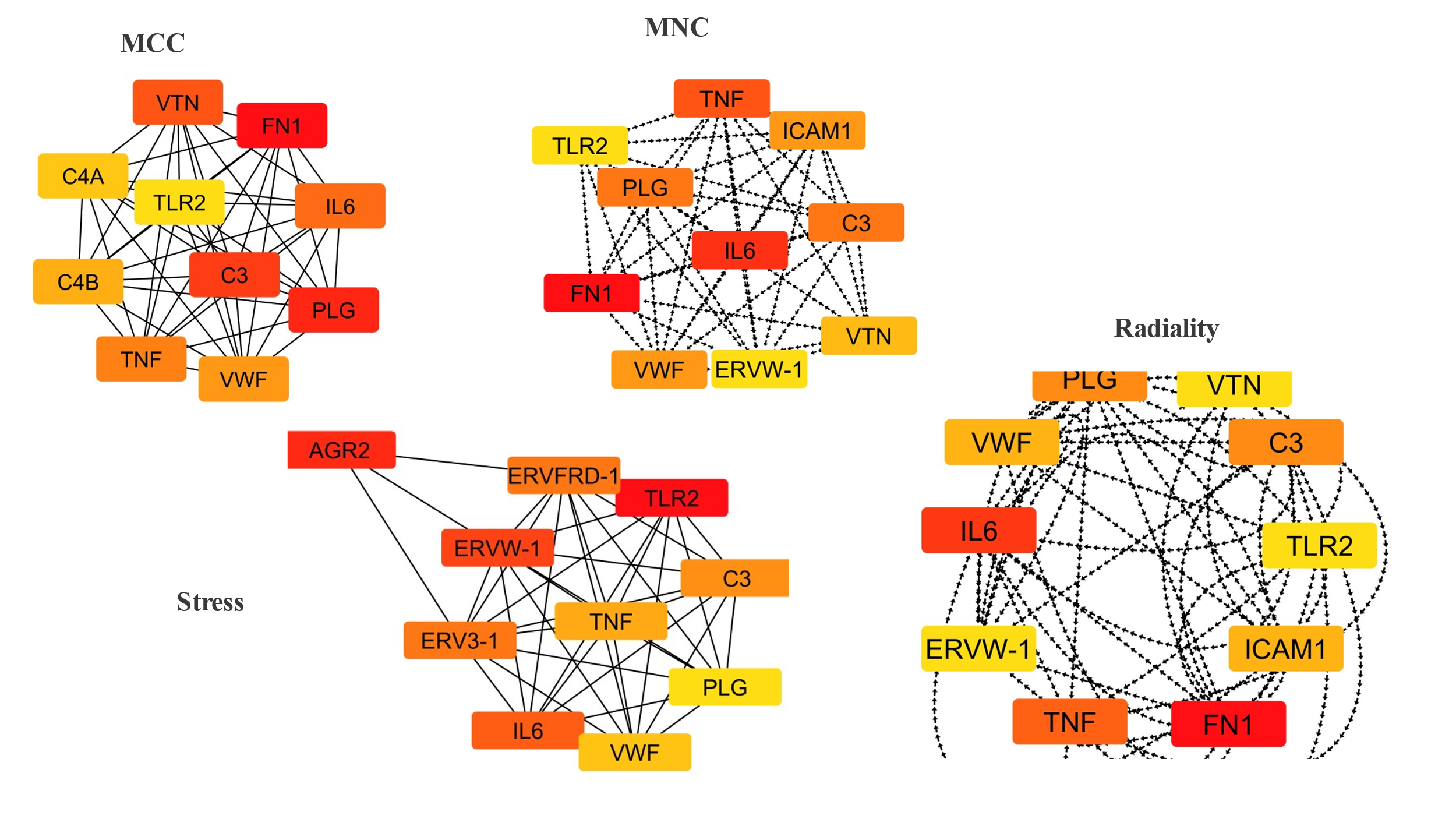


**Figure S6:** The occurrence of 10 hub genes by another different 4 methods in analysis of cytoHubba.

**Table S1:** GO enrichment entry**.**

| **Category** | **description** | **FDR value** | **term name** |
| --- | --- | --- | --- |
| GO Biological Process | Inflammatory response | 2.58E-12 | GO:0006954 |
| GO Biological Process | Biological process involved in interspecies interaction between organisms | 5.68E-11 | GO:0044419 |
| GO Biological Process | Defense response | 1.46E-10 | GO:0006952 |
| GO Biological Process | Response to stress | 8.45E-10 | GO:0006950 |
| GO Biological Process | Regulation of body fluid levels | 1.10E-09 | GO:0050878 |
| GO Biological Process | Immune system process | 2.20E-09 | GO:0002376 |
| GO Biological Process | Positive regulation of response to stimulus | 2.20E-09 | GO:0048584 |
| GO Biological Process | Regulation of cell adhesion | 2.26E-09 | GO:0030155 |
| GO Biological Process | Response to bacterium | 2.96E-09 | GO:0009617 |
| GO Biological Process | Negative regulation of endopeptidase activity | 2.96E-09 | GO:0010951 |
| GO Biological Process | Response to other organism | 2.96E-09 | GO:0051707 |
| GO Biological Process | Negative regulation of proteolysis | 3.34E-09 | GO:0045861 |
| GO Biological Process | Positive regulation of response to external stimulus | 3.96E-09 | GO:0032103 |
| GO Biological Process | Negative regulation of biological process | 4.11E-09 | GO:0048519 |
| GO Biological Process | Humoral immune response | 5.18E-09 | GO:0006959 |
| GO Biological Process | Defense response to other organism | 2.96E-08 | GO:0098542 |
| GO Biological Process | Wound healing | 4.86E-08 | GO:0042060 |
| GO Biological Process | Regulation of peptidase activity | 4.89E-08 | GO:0052547 |
| GO Biological Process | Response to external stimulus | 5.00E-08 | GO:0009605 |
| GO Biological Process | Negative regulation of protein metabolic process | 5.00E-08 | GO:0051248 |
| GO Biological Process | Blood coagulation | 5.52E-08 | GO:0007596 |
| GO Biological Process | Negative regulation of wound healing | 8.92E-08 | GO:0061045 |
| GO Biological Process | Positive regulation of cell adhesion | 9.06E-08 | GO:0045785 |
| GO Biological Process | Negative regulation of fibrinolysis | 1.16E-07 | GO:0051918 |
| GO Biological Process | Immune response | 1.23E-07 | GO:0006955 |
| GO Biological Process | Regulation of response to external stimulus | 1.49E-07 | GO:0032101 |
| GO Biological Process | Cell activation | 3.11E-07 | GO:0001775 |
| GO Biological Process | Regulation of response to stimulus | 3.43E-07 | GO:0048583 |
| GO Biological Process | Negative regulation of multicellular organismal process | 3.43E-07 | GO:0051241 |
| GO Biological Process | Negative regulation of blood coagulation | 3.58E-07 | GO:0030195 |
| GO Biological Process | Response to molecule of bacterial origin | 4.39E-07 | GO:0002237 |
| GO Biological Process | Multicellular organismal process | 5.03E-07 | GO:0032501 |
| GO Biological Process | Regulation of proteolysis | 5.20E-07 | GO:0030162 |
| GO Biological Process | Regulation of molecular function | 6.15E-07 | GO:0065009 |
| GO Biological Process | Regulation of phagocytosis | 7.84E-07 | GO:0050764 |
| GO Biological Process | Negative regulation of catalytic activity | 8.07E-07 | GO:0043086 |
| GO Biological Process | Immune effector process | 1.14E-06 | GO:0002252 |
| GO Biological Process | Regulation of response to stress | 1.30E-06 | GO:0080134 |
| GO Biological Process | Regulation of protein metabolic process | 1.32E-06 | GO:0051246 |
| GO Biological Process | Negative regulation of response to external stimulus | 1.44E-06 | GO:0032102 |
| GO Biological Process | Regulation of catalytic activity | 1.54E-06 | GO:0050790 |
| GO Biological Process | Regulation of hydrolase activity | 1.74E-06 | GO:0051336 |
| GO Biological Process | Regulation of immune system process | 2.19E-06 | GO:0002682 |
| GO Biological Process | Regulation of biological quality | 2.19E-06 | GO:0065008 |
| GO Biological Process | Positive regulation of cellular process | 2.45E-06 | GO:0048522 |
| GO Biological Process | Defense response to bacterium | 2.64E-06 | GO:0042742 |
| GO Biological Process | Negative regulation of macromolecule metabolic process | 2.65E-06 | GO:0010605 |
| GO Biological Process | Leukocyte mediated immunity | 2.72E-06 | GO:0002443 |
| GO Biological Process | Anatomical structure morphogenesis | 3.15E-06 | GO:0009653 |
| GO Biological Process | Regulation of cytokine production | 4.02E-06 | GO:0001817 |
| GO Biological Process | Positive regulation of signal transduction | 4.09E-06 | GO:0009967 |
| GO Biological Process | Acute inflammatory response | 4.31E-06 | GO:0002526 |
| GO Biological Process | Positive regulation of inflammatory response | 4.59E-06 | GO:0050729 |
| GO Biological Process | Negative regulation of molecular function | 6.19E-06 | GO:0044092 |
| GO Biological Process | Acute-phase response | 8.05E-06 | GO:0006953 |
| GO Biological Process | Response to stimulus | 8.16E-06 | GO:0050896 |
| GO Biological Process | Leukocyte migration | 8.20E-06 | GO:0050900 |
| GO Biological Process | Negative regulation of nitrogen compound metabolic process | 9.09E-06 | GO:0051172 |
| GO Biological Process | Anatomical structure development | 9.12E-06 | GO:0048856 |
| GO Biological Process | Biological process involved in symbiotic interaction | 9.75E-06 | GO:0044403 |
| GO Biological Process | Platelet activation | 1.11E-05 | GO:0030168 |
| GO Biological Process | Developmental process | 1.50E-05 | GO:0032502 |
| GO Biological Process | Regulation of phosphorylation | 1.60E-05 | GO:0042325 |
| GO Biological Process | Biological process involved in interaction with symbiont | 1.92E-05 | GO:0051702 |
| GO Biological Process | Positive regulation of immune system process | 1.93E-05 | GO:0002684 |
| GO Biological Process | Positive regulation of multicellular organismal process | 2.08E-05 | GO:0051240 |
| GO Biological Process | Fibrinolysis | 2.17E-05 | GO:0042730 |
| GO Biological Process | Cell migration | 2.62E-05 | GO:0016477 |
| GO Biological Process | Regulation of protein phosphorylation | 2.87E-05 | GO:0001932 |
| GO Biological Process | Positive regulation of locomotion | 2.87E-05 | GO:0040017 |
| GO Biological Process | Response to lipopolysaccharide | 3.67E-05 | GO:0032496 |
| GO Biological Process | Regulation of localization | 4.01E-05 | GO:0032879 |
| GO Biological Process | Neutrophil mediated immunity | 5.30E-05 | GO:0002446 |
| GO Biological Process | Myeloid leukocyte differentiation | 5.70E-05 | GO:0002573 |
| GO Biological Process | Regulation of multicellular organismal process | 6.05E-05 | GO:0051239 |
| GO Biological Process | Positive regulation of acute inflammatory response | 6.66E-05 | GO:0002675 |
| GO Biological Process | Regulation of cell-cell adhesion | 6.80E-05 | GO:0022407 |
| GO Biological Process | Regulation of tissue remodeling | 8.39E-05 | GO:0034103 |
| GO Biological Process | Positive regulation of phagocytosis | 8.39E-05 | GO:0050766 |
| GO Biological Process | Killing by host of symbiont cells | 8.39E-05 | GO:0051873 |
| GO Biological Process | Regulation of cell activation | 8.96E-05 | GO:0050865 |
| GO Biological Process | Antimicrobial humoral response | 9.34E-05 | GO:0019730 |
| GO Biological Process | Multicellular organism development | 1.00E-04 | GO:0007275 |
| GO Biological Process | Cell differentiation | 1.00E-04 | GO:0030154 |
| GO Biological Process | System development | 1.10E-04 | GO:0048731 |
| GO Biological Process | Regulation of inflammatory response | 1.10E-04 | GO:0050727 |
| GO Biological Process | Regulation of transport | 1.20E-04 | GO:0051049 |
| GO Biological Process | Positive regulation of cell migration | 1.30E-04 | GO:0030335 |
| GO Biological Process | Myeloid cell differentiation | 1.40E-04 | GO:0030099 |
| GO Biological Process | Leukocyte differentiation | 1.60E-04 | GO:0002521 |
| GO Biological Process | Positive regulation of apoptotic cell clearance | 1.60E-04 | GO:2000427 |
| GO Biological Process | Regulation of signal transduction | 1.70E-04 | GO:0009966 |
| GO Biological Process | Regulation of cellular component organization | 1.70E-04 | GO:0051128 |
| GO Biological Process | Regulation of vesicle-mediated transport | 1.80E-04 | GO:0060627 |
| GO Biological Process | Extracellular matrix organization | 1.90E-04 | GO:0030198 |
| GO Biological Process | Positive regulation of transport | 1.90E-04 | GO:0051050 |
| GO Biological Process | Neutrophil-mediated killing of bacterium | 2.00E-04 | GO:0070944 |
| GO Biological Process | Regulation of chemokine production | 2.10E-04 | GO:0032642 |
| GO Biological Process | Positive regulation of gene expression | 2.20E-04 | GO:0010628 |
| GO Biological Process | Leukocyte activation | 2.20E-04 | GO:0045321 |
| GO Biological Process | Negative regulation of response to stimulus | 2.20E-04 | GO:0048585 |
| GO Biological Process | Tissue remodeling | 2.60E-04 | GO:0048771 |
| GO Biological Process | Cellular extravasation | 2.70E-04 | GO:0045123 |
| GO Biological Process | Regulation of cell death | 2.80E-04 | GO:0010941 |
| GO Biological Process | Positive regulation of tissue remodeling | 3.10E-04 | GO:0034105 |
| GO Biological Process | Regulation of biological process | 3.10E-04 | GO:0050789 |
| GO Biological Process | Detection of molecule of bacterial origin | 3.70E-04 | GO:0032490 |
| GO Biological Process | Positive regulation of intracellular signal transduction | 3.70E-04 | GO:1902533 |
| GO Biological Process | Positive regulation of cell-cell adhesion | 4.20E-04 | GO:0022409 |
| GO Biological Process | Cellular response to molecule of bacterial origin | 4.20E-04 | GO:0071219 |
| GO Biological Process | Regulation of lipid storage | 4.50E-04 | GO:0010883 |
| GO Biological Process | Mononuclear cell migration | 4.60E-04 | GO:0071674 |
| GO Biological Process | Regulation of locomotion | 4.90E-04 | GO:0040012 |
| GO Biological Process | Positive regulation of phosphorylation | 4.90E-04 | GO:0042327 |
| GO Biological Process | Animal organ development | 5.20E-04 | GO:0048513 |
| GO Biological Process | Positive regulation of cytokine production | 5.30E-04 | GO:0001819 |
| GO Biological Process | Regulation of platelet activation | 5.30E-04 | GO:0010543 |
| GO Biological Process | Hemopoiesis | 5.60E-04 | GO:0030097 |
| GO Biological Process | Osteoclast differentiation | 5.70E-04 | GO:0030316 |
| GO Biological Process | Regulation of immune response | 5.70E-04 | GO:0050776 |
| GO Biological Process | Response to chemical | 6.80E-04 | GO:0042221 |
| GO Biological Process | Positive regulation of immune response | 6.80E-04 | GO:0050778 |
| GO Biological Process | Cell killing | 7.10E-04 | GO:0001906 |
| GO Biological Process | Defense response to Gram-positive bacterium | 7.10E-04 | GO:0050830 |
| GO Biological Process | Regulation of cell development | 7.10E-04 | GO:0060284 |
| GO Biological Process | Positive regulation of opsonization | 7.10E-04 | GO:1903028 |
| GO Biological Process | Negative regulation of miRNA maturation | 7.10E-04 | GO:1903799 |
| GO Biological Process | Response to organic substance | 7.30E-04 | GO:0010033 |
| GO Biological Process | Chemotaxis | 7.80E-04 | GO:0006935 |
| GO Biological Process | Positive regulation of homotypic cell-cell adhesion | 7.80E-04 | GO:0034112 |
| GO Biological Process | Positive regulation of developmental process | 8.00E-04 | GO:0051094 |
| GO Biological Process | Regulation of leukocyte cell-cell adhesion | 8.20E-04 | GO:1903037 |
| GO Biological Process | Macrophage activation | 8.40E-04 | GO:0042116 |
| GO Biological Process | Myeloid leukocyte migration | 8.80E-04 | GO:0097529 |
| GO Biological Process | Positive regulation of interleukin-8 production | 9.70E-04 | GO:0032757 |
| GO Biological Process | Positive regulation of tyrosine phosphorylation of STAT protein | 9.70E-04 | GO:0042531 |
| GO Biological Process | Regulation of immune effector process | 0.001 | GO:0002697 |
| GO Biological Process | Regulation of cell migration | 0.0011 | GO:0030334 |
| GO Biological Process | Negative regulation of nervous system development | 0.0011 | GO:0051961 |
| GO Biological Process | Regulation of multicellular organismal development | 0.0011 | GO:2000026 |
| GO Biological Process | Myeloid leukocyte activation | 0.0012 | GO:0002274 |
| GO Biological Process | Regulation of peptidyl-tyrosine phosphorylation | 0.0012 | GO:0050730 |
| GO Biological Process | Positive regulation of protein phosphorylation | 0.0013 | GO:0001934 |
| GO Biological Process | Positive regulation of immune effector process | 0.0013 | GO:0002699 |
| GO Biological Process | Blood circulation | 0.0013 | GO:0008015 |
| GO Biological Process | Regeneration | 0.0013 | GO:0031099 |
| GO Biological Process | Positive regulation of chemokine production | 0.0013 | GO:0032722 |
| GO Biological Process | Regulation of cell population proliferation | 0.0013 | GO:0042127 |
| GO Biological Process | Regulation of macromolecule metabolic process | 0.0013 | GO:0060255 |
| GO Biological Process | Tissue development | 0.0017 | GO:0009888 |
| GO Biological Process | Regulation of apoptotic process | 0.0017 | GO:0042981 |
| GO Biological Process | Regulation of intracellular signal transduction | 0.0017 | GO:1902531 |
| GO Biological Process | Negative regulation of cytokine production | 0.0018 | GO:0001818 |
| GO Biological Process | Adaptive immune response based on somatic recombination of immune receptors built from immunoglobulin superfamily domains | 0.0018 | GO:0002460 |
| GO Biological Process | Regulation of leukocyte activation | 0.0018 | GO:0002694 |
| GO Biological Process | Positive regulation of fractalkine production | 0.0018 | GO:0032724 |
| GO Biological Process | Detection of triacyl bacterial lipopeptide | 0.0018 | GO:0042495 |
| GO Biological Process | Regulation of lipid localization | 0.0018 | GO:1905952 |
| GO Biological Process | Response to gold nanoparticle | 0.0018 | GO:1990268 |
| GO Biological Process | Negative regulation of cell adhesion | 0.0019 | GO:0007162 |
| GO Biological Process | Positive regulation of osteoclast differentiation | 0.0019 | GO:0045672 |
| GO Biological Process | Negative regulation of cellular process | 0.0019 | GO:0048523 |
| GO Biological Process | Cell surface receptor signaling pathway | 0.002 | GO:0007166 |
| GO Biological Process | Response to cytokine | 0.002 | GO:0034097 |
| GO Biological Process | Negative regulation of cell death | 0.002 | GO:0060548 |
| GO Biological Process | Protein kinase C-activating G protein-coupled receptor signaling pathway | 0.0021 | GO:0007205 |
| GO Biological Process | Regulation of ERK1 and ERK2 cascade | 0.0022 | GO:0070372 |
| GO Biological Process | Positive regulation of cellular component organization | 0.0025 | GO:0051130 |
| GO Biological Process | Regulation of blood vessel endothelial cell migration | 0.0026 | GO:0043535 |
| GO Biological Process | Cellular response to triacyl bacterial lipopeptide | 0.0027 | GO:0071727 |
| GO Biological Process | Liver regeneration | 0.0027 | GO:0097421 |
| GO Biological Process | Microglial cell activation | 0.0029 | GO:0001774 |
| GO Biological Process | Positive regulation of peptidyl-tyrosine phosphorylation | 0.0032 | GO:0050731 |
| GO Biological Process | Positive regulation of molecular function | 0.0033 | GO:0044093 |
| GO Biological Process | Defense response to Gram-negative bacterium | 0.0033 | GO:0050829 |
| GO Biological Process | Cellular response to lipopolysaccharide | 0.0033 | GO:0071222 |
| GO Biological Process | Positive regulation of interleukin-6 production | 0.0034 | GO:0032755 |
| GO Biological Process | Regulation of MAPK cascade | 0.0034 | GO:0043408 |
| GO Biological Process | Response to tumor necrosis factor | 0.0035 | GO:0034612 |
| GO Biological Process | Positive regulation of cell differentiation | 0.0035 | GO:0045597 |
| GO Biological Process | Regulation of gliogenesis | 0.0037 | GO:0014013 |
| GO Biological Process | Positive regulation of tumor necrosis factor production | 0.0037 | GO:0032760 |
| GO Biological Process | Regulation of developmental process | 0.0038 | GO:0050793 |
| GO Biological Process | Gland morphogenesis | 0.0041 | GO:0022612 |
| GO Biological Process | Regulation of collagen biosynthetic process | 0.0041 | GO:0032965 |
| GO Biological Process | Positive regulation of apoptotic process | 0.0042 | GO:0043065 |
| GO Biological Process | Cell chemotaxis | 0.0043 | GO:0060326 |
| GO Biological Process | Response to mechanical stimulus | 0.0046 | GO:0009612 |
| GO Biological Process | Cellular response to cytokine stimulus | 0.0048 | GO:0071345 |
| GO Biological Process | Positive regulation of adaptive immune response based on somatic recombination of immune receptors built from immunoglobulin superfamily domains | 0.0049 | GO:0002824 |
| GO Biological Process | Regulation of cell-substrate adhesion | 0.0049 | GO:0010810 |
| GO Biological Process | Adaptive immune response | 0.0051 | GO:0002250 |
| GO Biological Process | Complement activation, classical pathway | 0.0052 | GO:0006958 |
| GO Biological Process | Anatomical structure formation involved in morphogenesis | 0.0054 | GO:0048646 |
| GO Biological Process | Regulation of cellular process | 0.0056 | GO:0050794 |
| GO Biological Process | Positive regulation of lymphocyte mediated immunity | 0.0057 | GO:0002708 |
| GO Biological Process | Regulation of neurogenesis | 0.0057 | GO:0050767 |
| GO Biological Process | Positive regulation of immunoglobulin mediated immune response | 0.0058 | GO:0002891 |
| GO Biological Process | Formation of primary germ layer | 0.0061 | GO:0001704 |
| GO Biological Process | Monocyte chemotaxis | 0.0061 | GO:0002548 |
| GO Biological Process | Cell development | 0.0061 | GO:0048468 |
| GO Biological Process | Regulation of T cell activation | 0.0062 | GO:0050863 |
| GO Biological Process | Regulation of calcidiol 1-monooxygenase activity | 0.0062 | GO:0060558 |
| GO Biological Process | Positive regulation of apoptotic DNA fragmentation | 0.0062 | GO:1902512 |
| GO Biological Process | Regulation of activated T cell proliferation | 0.0067 | GO:0046006 |
| GO Biological Process | Positive regulation of receptor signaling pathway via JAK-STAT | 0.0067 | GO:0046427 |
| GO Biological Process | Regulation of leukocyte mediated immunity | 0.0068 | GO:0002703 |
| GO Biological Process | Bone remodeling | 0.0071 | GO:0046849 |
| GO Biological Process | Vascular endothelial growth factor production | 0.0075 | GO:0010573 |
| GO Biological Process | Positive regulation of cell-substrate adhesion | 0.0075 | GO:0010811 |
| GO Biological Process | Positive regulation of fever generation | 0.0075 | GO:0031622 |
| GO Biological Process | Regulation of vitamin D biosynthetic process | 0.0075 | GO:0060556 |
| GO Biological Process | Cellular response to nicotine | 0.0075 | GO:0071316 |
| GO Biological Process | Negative regulation of lipid localization | 0.0075 | GO:1905953 |
| GO Biological Process | Response to nicotine | 0.0077 | GO:0035094 |
| GO Biological Process | Positive regulation of protein metabolic process | 0.0082 | GO:0051247 |
| GO Biological Process | Regulation of nitrogen compound metabolic process | 0.0087 | GO:0051171 |
| GO Biological Process | Positive regulation of interleukin-18 production | 0.009 | GO:0032741 |
| GO Biological Process | Response to oxygen-containing compound | 0.0097 | GO:1901700 |
| GO Biological Process | Protein kinase B signaling | 0.0099 | GO:0043491 |
| GO Biological Process | Gland development | 0.0099 | GO:0048732 |
| GO Biological Process | Neurogenesis | 0.0101 | GO:0022008 |
| GO Biological Process | Endoderm formation | 0.0103 | GO:0001706 |
| GO Biological Process | Regulation of smooth muscle cell proliferation | 0.0105 | GO:0048660 |
| GO Biological Process | Positive regulation of DNA-binding transcription factor activity | 0.0105 | GO:0051091 |
| GO Biological Process | Opsonization | 0.0107 | GO:0008228 |
| GO Biological Process | Negative regulation of neurogenesis | 0.0107 | GO:0050768 |
| GO Biological Process | Positive regulation of metabolic process | 0.011 | GO:0009893 |
| GO Biological Process | Tumor necrosis factor-mediated signaling pathway | 0.0111 | GO:0033209 |
| GO Biological Process | Negative regulation of transport | 0.0111 | GO:0051051 |
| GO Biological Process | Regulation of cytokine production involved in inflammatory response | 0.0111 | GO:1900015 |
| GO Biological Process | Regulation of cell differentiation | 0.0112 | GO:0045595 |
| GO Biological Process | Positive regulation of leukocyte cell-cell adhesion | 0.0113 | GO:1903039 |
| GO Biological Process | JNK cascade | 0.0115 | GO:0007254 |
| GO Biological Process | Leukocyte chemotaxis | 0.012 | GO:0030595 |
| GO Biological Process | Positive regulation of leukocyte migration | 0.0126 | GO:0002687 |
| GO Biological Process | Positive regulation of macromolecule metabolic process | 0.0126 | GO:0010604 |
| GO Biological Process | Regulation of primary metabolic process | 0.0126 | GO:0080090 |
| GO Biological Process | Positive regulation of protein localization to plasma membrane | 0.013 | GO:1903078 |
| GO Biological Process | Toll-like receptor signaling pathway | 0.0135 | GO:0002224 |
| GO Biological Process | Positive regulation of interleukin-1 beta production | 0.0135 | GO:0032731 |
| GO Biological Process | Regulation of angiogenesis | 0.0137 | GO:0045765 |
| GO Biological Process | Defense response to fungus | 0.014 | GO:0050832 |
| GO Biological Process | Lymphocyte mediated immunity | 0.0147 | GO:0002449 |
| GO Biological Process | Tube development | 0.0155 | GO:0035295 |
| GO Biological Process | Cytolysis by host of symbiont cells | 0.016 | GO:0051838 |
| GO Biological Process | Positive regulation of platelet aggregation | 0.016 | GO:1901731 |
| GO Biological Process | Positive regulation of gliogenesis | 0.0161 | GO:0014015 |
| GO Biological Process | Negative regulation of apoptotic process | 0.0164 | GO:0043066 |
| GO Biological Process | Positive regulation of cell development | 0.0166 | GO:0010720 |
| GO Biological Process | Intrinsic apoptotic signaling pathway | 0.0166 | GO:0097193 |
| GO Biological Process | I-kappaB kinase/NF-kappaB signaling | 0.0172 | GO:0007249 |
| GO Biological Process | Negative regulation of gene expression | 0.0172 | GO:0010629 |
| GO Biological Process | Circulatory system development | 0.0173 | GO:0072359 |
| GO Biological Process | Calcium-mediated signaling | 0.0174 | GO:0019722 |
| GO Biological Process | Positive regulation of inflammatory response to antigenic stimulus | 0.0175 | GO:0002863 |
| GO Biological Process | Epithelial cell proliferation | 0.0175 | GO:0050673 |
| GO Biological Process | Trophoblast giant cell differentiation | 0.0175 | GO:0060707 |
| GO Biological Process | Cellular response to tumor necrosis factor | 0.0193 | GO:0071356 |
| GO Biological Process | Regulation of anatomical structure morphogenesis | 0.0194 | GO:0022603 |
| GO Biological Process | Apoptotic signaling pathway | 0.0194 | GO:0097190 |
| GO Biological Process | Positive regulation of kinase activity | 0.0195 | GO:0033674 |
| GO Biological Process | Muscle structure development | 0.0202 | GO:0061061 |
| GO Biological Process | Regulation of glial cell differentiation | 0.0207 | GO:0045685 |
| GO Biological Process | Negative regulation of developmental process | 0.0207 | GO:0051093 |
| GO Biological Process | Intrinsic apoptotic signaling pathway in response to DNA damage | 0.0212 | GO:0008630 |
| GO Biological Process | Positive regulation of leukocyte differentiation | 0.0212 | GO:1902107 |
| GO Biological Process | Mammary gland epithelial cell proliferation | 0.0213 | GO:0033598 |
| GO Biological Process | Positive regulation of neuroinflammatory response | 0.0213 | GO:0150078 |
| GO Biological Process | Negative regulation of cell population proliferation | 0.0216 | GO:0008285 |
| GO Biological Process | Regulation of immunoglobulin production | 0.0217 | GO:0002637 |
| GO Biological Process | Myotube differentiation | 0.0217 | GO:0014902 |
| GO Biological Process | Positive regulation of cell population proliferation | 0.0218 | GO:0008284 |
| GO Biological Process | Regulation of peptide hormone secretion | 0.0223 | GO:0090276 |
| GO Biological Process | Regulation of T cell proliferation | 0.0231 | GO:0042129 |
| GO Biological Process | Regulation of establishment of endothelial barrier | 0.0234 | GO:1903140 |
| GO Biological Process | Positive regulation of phosphatidylinositol 3-kinase signaling | 0.0236 | GO:0014068 |
| GO Biological Process | Regulation of blood pressure | 0.0245 | GO:0008217 |
| GO Biological Process | Regulation of hormone levels | 0.0249 | GO:0010817 |
| GO Biological Process | Regulation of cellular component biogenesis | 0.0253 | GO:0044087 |
| GO Biological Process | Negative regulation of cell-cell adhesion | 0.0256 | GO:0022408 |
| GO Biological Process | Regulation of lipid metabolic process | 0.0258 | GO:0019216 |
| GO Biological Process | Intracellular signal transduction | 0.0278 | GO:0035556 |
| GO Biological Process | Positive regulation of cellular metabolic process | 0.0279 | GO:0031325 |
| GO Biological Process | Positive regulation of glial cell proliferation | 0.0279 | GO:0060252 |
| GO Biological Process | Positive regulation of smooth muscle cell proliferation | 0.028 | GO:0048661 |
| GO Biological Process | Positive regulation of transcription by RNA polymerase II | 0.0284 | GO:0045944 |
| GO Biological Process | Regulation of cell junction assembly | 0.0285 | GO:1901888 |
| GO Biological Process | Response to virus | 0.0286 | GO:0009615 |
| GO Biological Process | Animal organ morphogenesis | 0.029 | GO:0009887 |
| GO Biological Process | Striated muscle cell differentiation | 0.0299 | GO:0051146 |
| GO Biological Process | Signaling | 0.0307 | GO:0023052 |
| GO Biological Process | Positive regulation of nitrogen compound metabolic process | 0.0324 | GO:0051173 |
| GO Biological Process | Cytokine-mediated signaling pathway | 0.0329 | GO:0019221 |
| GO Biological Process | Regulation of kinase activity | 0.0329 | GO:0043549 |
| GO Biological Process | Regulation of cell-cell adhesion mediated by cadherin | 0.0329 | GO:2000047 |
| GO Biological Process | Regulation of bicellular tight junction assembly | 0.0329 | GO:2000810 |
| GO Biological Process | Regulation of secretion by cell | 0.0339 | GO:1903530 |
| GO Biological Process | Positive regulation of ERK1 and ERK2 cascade | 0.0342 | GO:0070374 |
| GO Biological Process | MyD88-dependent toll-like receptor signaling pathway | 0.0354 | GO:0002755 |
| GO Biological Process | Signal transduction | 0.0354 | GO:0007165 |
| GO Biological Process | Negative regulation of lipid storage | 0.0354 | GO:0010888 |
| GO Biological Process | Positive regulation of amyloid-beta formation | 0.0354 | GO:1902004 |
| GO Biological Process | Negative regulation of cell activation | 0.0367 | GO:0050866 |
| GO Biological Process | Apoptotic process | 0.0368 | GO:0006915 |
| GO Biological Process | MAPK cascade | 0.0371 | GO:0000165 |
| GO Biological Process | Positive regulation of JNK cascade | 0.0372 | GO:0046330 |
| GO Biological Process | Positive regulation of calcium ion transmembrane transport | 0.0372 | GO:1904427 |
| GO Biological Process | Positive regulation of leukocyte activation | 0.0376 | GO:0002696 |
| GO Biological Process | Positive regulation of humoral immune response | 0.0376 | GO:0002922 |
| GO Biological Process | Regulation of systemic arterial blood pressure by renin-angiotensin | 0.0376 | GO:0003081 |
| GO Biological Process | Negative regulation of phagocytosis | 0.0376 | GO:0050765 |
| GO Biological Process | Embryo development | 0.038 | GO:0009790 |
| GO Biological Process | Cell communication | 0.0386 | GO:0007154 |
| GO Biological Process | Positive regulation of endocytosis | 0.0396 | GO:0045807 |
| GO Biological Process | Cellular response to oxygen-containing compound | 0.0396 | GO:1901701 |
| GO Biological Process | Negative regulation of heart contraction | 0.0399 | GO:0045822 |
| GO Biological Process | Positive regulation of calcineurin-NFAT signaling cascade | 0.0399 | GO:0070886 |
| GO Biological Process | Positive regulation of leukocyte adhesion to vascular endothelial cell | 0.0399 | GO:1904996 |
| GO Biological Process | Regulation of systemic arterial blood pressure | 0.0402 | GO:0003073 |
| GO Biological Process | Positive regulation of neurogenesis | 0.0406 | GO:0050769 |
| GO Biological Process | Epithelium development | 0.0415 | GO:0060429 |
| GO Biological Process | Positive regulation of lipid storage | 0.0423 | GO:0010884 |
| GO Biological Process | Negative regulation of chemokine production | 0.0423 | GO:0032682 |
| GO Biological Process | Blood coagulation, fibrin clot formation | 0.0423 | GO:0072378 |
| GO Biological Process | Positive regulation of cytokine production involved in inflammatory response | 0.0423 | GO:1900017 |
| GO Biological Process | Negative regulation of angiogenesis | 0.0427 | GO:0016525 |
| GO Biological Process | Positive regulation of transmembrane transport | 0.0451 | GO:0034764 |
| GO Biological Process | Regulation of epithelial cell apoptotic process | 0.0455 | GO:1904035 |
| GO Biological Process | Cellular response to chemical stimulus | 0.0461 | GO:0070887 |
| GO Biological Process | Positive regulation of collagen biosynthetic process | 0.0472 | GO:0032967 |
| GO Biological Process | Positive regulation of T cell proliferation | 0.0472 | GO:0042102 |
| GO Biological Process | Epithelial cell differentiation | 0.0475 | GO:0030855 |
| GO Biological Process | Regulation of protein localization | 0.0486 | GO:0032880 |
| GO Cellular Component | Blood microparticle | 2.42E-12 | GO:0072562 |
| GO Cellular Component | Secretory granule lumen | 6.03E-11 | GO:0034774 |
| GO Cellular Component | Collagen-containing extracellular matrix | 4.46E-10 | GO:0062023 |
| GO Cellular Component | Extracellular space | 2.21E-08 | GO:0005615 |
| GO Cellular Component | Platelet alpha granule lumen | 2.71E-08 | GO:0031093 |
| GO Cellular Component | Extracellular region | 8.61E-08 | GO:0005576 |
| GO Cellular Component | Secretory granule | 1.64E-07 | GO:0030141 |
| GO Cellular Component | Cell periphery | 2.06E-07 | GO:0071944 |
| GO Cellular Component | Endoplasmic reticulum lumen | 1.70E-06 | GO:0005788 |
| GO Cellular Component | Extracellular exosome | 4.56E-06 | GO:0070062 |
| GO Cellular Component | Endomembrane system | 4.86E-06 | GO:0012505 |
| GO Cellular Component | Azurophil granule lumen | 4.86E-06 | GO:0035578 |
| GO Cellular Component | Membrane raft | 2.40E-05 | GO:0045121 |
| GO Cellular Component | Vesicle | 9.32E-05 | GO:0031982 |
| GO Cellular Component | Plasma membrane | 1.10E-04 | GO:0005886 |
| GO Cellular Component | Cytoplasmic vesicle | 9.10E-04 | GO:0031410 |
| GO Cellular Component | Toll-like receptor 1-Toll-like receptor 2 protein complex | 0.0016 | GO:0035354 |
| GO Cellular Component | Classical-complement-pathway C3/C5 convertase complex | 0.0038 | GO:0005601 |
| GO Cellular Component | Cell surface | 0.004 | GO:0009986 |
| GO Cellular Component | Endoplasmic reticulum | 0.0071 | GO:0005783 |
| GO Cellular Component | Intracellular organelle lumen | 0.008 | GO:0070013 |
| GO Cellular Component | Fibrinogen complex | 0.0096 | GO:0005577 |
| GO Cellular Component | Lysosome | 0.0373 | GO:0005764 |
| GO Molecular Function | Signaling receptor binding | 1.45E-12 | GO:0005102 |
| GO Molecular Function | Peptidase regulator activity | 3.18E-11 | GO:0061134 |
| GO Molecular Function | Endopeptidase inhibitor activity | 1.03E-10 | GO:0004866 |
| GO Molecular Function | Glycosaminoglycan binding | 6.55E-11 | GO:0005539 |
| GO Molecular Function | Heparin binding | 8.44E-11 | GO:0008201 |
| GO Molecular Function | Enzyme inhibitor activity | 5.93E-09 | GO:0004857 |
| GO Molecular Function | Molecular function regulator activity | 8.94E-08 | GO:0098772 |
| GO Molecular Function | Protein binding | 1.83E-07 | GO:0005515 |
| GO Molecular Function | Protease binding | 3.75E-07 | GO:0002020 |
| GO Molecular Function | Serine-type endopeptidase inhibitor activity | 2.00E-06 | GO:0004867 |
| GO Molecular Function | Enzyme regulator activity | 4.23E-06 | GO:0030234 |
| GO Molecular Function | Collagen binding | 1.20E-05 | GO:0005518 |
| GO Molecular Function | Serine-type endopeptidase activity | 3.11E-05 | GO:0004252 |
| GO Molecular Function | Proteoglycan binding | 7.23E-05 | GO:0043394 |
| GO Molecular Function | Tumor necrosis factor receptor superfamily binding | 1.70E-04 | GO:0032813 |
| GO Molecular Function | Protein-containing complex binding | 1.70E-04 | GO:0044877 |

**Table S2:** KEGG enrichment entry.

| **Category** | **Description** | **FDR value** | **Term name** |
| --- | --- | --- | --- |
| KEGG Pathways | Complement and coagulation cascades | 9.57E-15 | hsa04610 |
| KEGG Pathways | Chagas disease | 5.49E-09 | hsa05142 |
| KEGG Pathways | Tuberculosis | 6.32E-09 | hsa05152 |
| KEGG Pathways | Pertussis | 1.93E-08 | hsa05133 |
| KEGG Pathways | Amoebiasis | 1.30E-07 | hsa05146 |
| KEGG Pathways | Legionellosis | 1.34E-07 | hsa05134 |
| KEGG Pathways | PI3K-Akt signaling pathway | 1.54E-06 | hsa04151 |
| KEGG Pathways | Influenza A | 1.91E-06 | hsa05164 |
| KEGG Pathways | AGE-RAGE signaling pathway in diabetic complications | 2.04E-06 | hsa04933 |
| KEGG Pathways | MAPK signaling pathway | 3.76E-06 | hsa04010 |
| KEGG Pathways | TNF signaling pathway | 3.76E-06 | hsa04668 |
| KEGG Pathways | Kaposi sarcoma-associated herpesvirus infection | 3.76E-06 | hsa05167 |
| KEGG Pathways | Inflammatory bowel disease | 4.76E-06 | hsa05321 |
| KEGG Pathways | Human T-cell leukemia virus 1 infection | 5.79E-06 | hsa05166 |
| KEGG Pathways | Rheumatoid arthritis | 2.05E-05 | hsa05323 |
| KEGG Pathways | *Staphylococcus aureus* infection | 2.27E-05 | hsa05150 |
| KEGG Pathways | African trypanosomiasis | 2.48E-05 | hsa05143 |
| KEGG Pathways | Systemic lupus erythematosus | 3.06E-05 | hsa05322 |
| KEGG Pathways | NF-kappa B signaling pathway | 3.88E-05 | hsa04064 |
| KEGG Pathways | Toll-like receptor signaling pathway | 3.88E-05 | hsa04620 |
| KEGG Pathways | C-type lectin receptor signaling pathway | 3.88E-05 | hsa04625 |
| KEGG Pathways | Pathogenic Escherichia coli infection | 3.88E-05 | hsa05130 |
| KEGG Pathways | Toxoplasmosis | 3.88E-05 | hsa05145 |
| KEGG Pathways | Epstein-Barr virus infection | 3.88E-05 | hsa05169 |
| KEGG Pathways | Malaria | 4.18E-05 | hsa05144 |
| KEGG Pathways | Human immunodeficiency virus 1 infection | 4.82E-05 | hsa05170 |
| KEGG Pathways | Human papillomavirus infection | 5.05E-05 | hsa05165 |
| KEGG Pathways | Salmonella infection | 5.27E-05 | hsa05132 |
| KEGG Pathways | Osteoclast differentiation | 6.00E-05 | hsa04380 |
| KEGG Pathways | Human cytomegalovirus infection | 6.06E-05 | hsa05163 |
| KEGG Pathways | Yersinia infection | 6.55E-05 | hsa05135 |
| KEGG Pathways | Pathways in cancer | 8.81E-05 | hsa05200 |
| KEGG Pathways | Measles | 9.82E-05 | hsa05162 |
| KEGG Pathways | Non-alcoholic fatty liver disease | 1.30E-04 | hsa04932 |
| KEGG Pathways | Leishmaniasis | 1.40E-04 | hsa05140 |
| KEGG Pathways | Hepatitis B | 1.80E-04 | hsa05161 |
| KEGG Pathways | IL-17 signaling pathway | 3.70E-04 | hsa04657 |
| KEGG Pathways | Antifolate resistance | 3.90E-04 | hsa01523 |
| KEGG Pathways | Viral protein interaction with cytokine and cytokine receptor | 4.10E-04 | hsa04061 |
| KEGG Pathways | Herpes simplex virus 1 infection | 4.10E-04 | hsa05168 |
| KEGG Pathways | Proteoglycans in cancer | 4.10E-04 | hsa05205 |
| KEGG Pathways | Neuroactive ligand-receptor interaction | 4.30E-04 | hsa04080 |
| KEGG Pathways | Th17 cell differentiation | 4.30E-04 | hsa04659 |
| KEGG Pathways | T cell receptor signaling pathway | 4.40E-04 | hsa04660 |
| KEGG Pathways | Graft-versus-host disease | 5.00E-04 | hsa05332 |
| KEGG Pathways | Insulin resistance | 5.20E-04 | hsa04931 |
| KEGG Pathways | Alzheimer disease | 5.70E-04 | hsa05010 |
| KEGG Pathways | Sphingolipid signaling pathway | 7.00E-04 | hsa04071 |
| KEGG Pathways | Natural killer cell mediated cytotoxicity | 7.80E-04 | hsa04650 |
| KEGG Pathways | Apoptosis | 0.001 | hsa04210 |
| KEGG Pathways | Fluid shear stress and atherosclerosis | 0.001 | hsa05418 |
| KEGG Pathways | Cytokine-cytokine receptor interaction | 0.0018 | hsa04060 |
| KEGG Pathways | Hepatitis C | 0.002 | hsa05160 |
| KEGG Pathways | Adipocytokine signaling pathway | 0.0025 | hsa04920 |
| KEGG Pathways | Th1 and Th2 cell differentiation | 0.0046 | hsa04658 |
| KEGG Pathways | PD-L1 expression and PD-1 checkpoint pathway in cancer | 0.0048 | hsa05235 |
| KEGG Pathways | ECM-receptor interaction | 0.0049 | hsa04512 |
| KEGG Pathways | Regulation of actin cytoskeleton | 0.0051 | hsa04810 |
| KEGG Pathways | Small cell lung cancer | 0.0053 | hsa05222 |
| KEGG Pathways | Shigellosis | 0.0058 | hsa05131 |
| KEGG Pathways | Apoptosis - multiple species | 0.0105 | hsa04215 |
| KEGG Pathways | Prion disease | 0.0111 | hsa05020 |
| KEGG Pathways | Allograft rejection | 0.0129 | hsa05330 |
| KEGG Pathways | Type I diabetes mellitus | 0.0156 | hsa04940 |
| KEGG Pathways | Phagosome | 0.0159 | hsa04145 |
| KEGG Pathways | Cellular senescence | 0.0186 | hsa04218 |
| KEGG Pathways | Intestinal immune network for IgA production | 0.0188 | hsa04672 |
| KEGG Pathways | MicroRNAs in cancer | 0.0212 | hsa05206 |
| KEGG Pathways | Transcriptional misregulation in cancer | 0.0255 | hsa05202 |
| KEGG Pathways | NOD-like receptor signaling pathway | 0.0259 | hsa04621 |
| KEGG Pathways | Axon guidance | 0.0264 | hsa04360 |
| KEGG Pathways | Amyotrophic lateral sclerosis | 0.0264 | hsa05014 |
| KEGG Pathways | Viral myocarditis | 0.0274 | hsa05416 |
| KEGG Pathways | Viral carcinogenesis | 0.0286 | hsa05203 |
| KEGG Pathways | Focal adhesion | 0.0334 | hsa04510 |
| KEGG Pathways | Cytosolic DNA-sensing pathway | 0.0334 | hsa04623 |
| KEGG Pathways | Epithelial cell signaling in Helicobacter pylori infection | 0.0355 | hsa05120 |
| KEGG Pathways | Acute myeloid leukemia | 0.0371 | hsa05221 |
| KEGG Pathways | Prolactin signaling pathway | 0.0377 | hsa04917 |
| KEGG Pathways | RIG-I-like receptor signaling pathway | 0.0383 | hsa04622 |
| KEGG Pathways | Ras signaling pathway | 0.0455 | hsa04014 |
| KEGG Pathways | B cell receptor signaling pathway | 0.0469 | hsa04662 |
|  |  |  |  |

**Table S3.** Docking results (S; kcal/mol) of the identified compounds (**1-25**) from *Aspergillus* sp and the co-crystallized ligands within the crystal structure of the targeted enzymes

| **NO** | **Compound** | **TLR2 (PDB ID: 6NIG)** | | **VWF (PDB: 1AUQ)** | | **TNF-α (PDB ID: 2AZ5)** | | **PBP-2a (PDB ID: 1VQQ)** | |
| --- | --- | --- | --- | --- | --- | --- | --- | --- | --- |
|  |  | S (kcal/mol) | RMSD (Å) | S (kcal/mol) | RMSD (Å) | S (kcal/mol) | RMSD (Å) | S (kcal/mol) | RMSD (Å) |
| **1** | **Aspergone M** | -5.198 | 0.778 | -5.527 | 1.528 | -4.370 | 1.4162439 | -5.49603 | 2.6505296 |
| **2** | **Aspilactonol D** | -5.108 | 1.056 | -7.539 | 1.359 | -4.641 | 1.6040268 | -5.52065 | 1.4802005 |
| **3** | **Methyl α-methyl-4-nitrobenzenepropanoate** | -5.489 | 1.473 | -5.494 | 1.103 | -4.369 | 0.70384413 | -5.54716 | 1.6898845 |
| **4** | **5-[(3*E*,5*E*)-3,5-nonadienyl]-1,3-benzenediol** | -5.373 | 1.325 | -6.341 | 1.477 | -4.906 | 1.8683133 | -5.5924 | 1.2646741 |
| **5** | **JBIR-75** | -5.392 | 1.420 | -5.038 | 1.317 | -4.448 | 0.94255471 | -5.14483 | 2.1160781 |
| **6** | **Asperitaconic acid A** | -5.416 | 1.077 | -5.996 | 1.665 | -4.593 | 1.0718758 | -5.79388 | 4.4041891 |
| **7** | **Puniceusine G** | -5.327 | 1.817 | -4.777 | 1.902 | -4.902 | 1.1492109 | -5.26549 | 1.3935241 |
| **8** | **9*α*-hydroxy-5*α*-drim-7-ene-6-one-11,12-olide** | -5.235 | 1.026 | -4.579 | 1.027 | -4.195 | 1.0741637 | -5.10592 | 1.332247 |
| **9** | **Ustusorane E** | -5.714 | 0.956 | -5.409 | 1.813 | -4.676 | 1.4801462 | -5.26239 | 1.2706242 |
| **10a** | **3-Hydroxy-6-methoxy-4-phenylquinolin-2(1H)-one** | -5.450 | 1.561 | -5.382 | 1.813 | -4.487 | 1.9538746 | -5.43536 | 2.7696371 |
| **10b** | **3-Methoxy-6-hydroxy-4-phenylquinolin-2(1H)-one** | -5.473 | 2.710 | -4.881 | 1.58 | -5.109 | 1.3526471 | -5.08607 | 5.3344893 |
| **11** | **Dihydrobipolaroxin B** | -5.214 | 2.555 | -4.871 | 1.761 | -4.436 | 1.6104553 | -5.70301 | 1.4183359 |
| **12** | **3 -Hydroxy4 -((2 R,6 R ) - 6 -(hydroxymethyl) -2,6 -dimethyltetrahydro - 2 H-pyran2 -yl) benzoic acid** | -5.546 | 1.627 | -5.138 | 1.023 | -4.416 | 0.82735395 | -5.82753 | 1.7296108 |
| **13** | **Versicone J** | -5.164 | 1.259 | -5.501 | 1.343 | -4.964 | 1.8478217 | -5.8159 | 1.7004805 |
| **14** | **Aspormisin A** | -6.583 | 2.761 | -6.379 | 1.510 | -4.870 | 1.980271 | -6.73174 | 1.7512854 |
| **15** | **Terreusinone** | -6.799 | 1.267 | -4.915 | 1.446 | -5.125 | 1.24523 | -6.25118 | 1.6283089 |
| **16** | **Aspergillone** | -6.364 | 0.885 | -6.177 | 1.948 | -5.327 | 1.0464309 | -6.46114 | 1.2318153 |
| **17a** | **Spiculisporic acid C** | -6.728 | 1.538 | -7.357 | 1.313 | -5.41263 | 1.1108854 | -6.2222 | 3.3007162 |
| **17b** | **Spiculisporic acid D** | -6.983 | 2.083 | -7.375 | 1.506 | -5.5001 | 1.7143834 | -6.45252 | 1.9721371 |
| **18** | **Protuboxepin G** | -6.493 | 1.375 | -6.118 | 1.089 | -4.94192 | 1.4453099 | -6.04662 | 1.5611236 |
| **19** | **Puniceusine E** | -7.061 | 1.165 | -5.127 | 1.796 | -5.32087 | 1.0818048 | -6.23427 | 1.6940231 |
| **20** | **Tryprostatin A** | -6.747 | 2.301 | -6.235 | 1.416 | -5.61435 | 1.1296334 | -7.02885 | 1.5908194 |
| **21** | **Puniceusine J** | -6.995 | 1.879 | -5.313 | 1.601 | -5.72362 | 1.885957 | -6.34786 | 1.4748894 |
| **22** | **(5*S*,6*S*)-16,17-Dihydroophiobolin H** | -7.082 | 2.036 | -6.047 | 1.949 | -5.24763 | 1.8130654 | -6.85122 | 1.7428789 |
| **23** | **Sclerotiotide E** | -7.386 | 1.608 | -6.827 | 1.293 | -5.93256 | 1.1446735 | -6.7511 | 1.2240716 |
| **24** | **6-epi-Avrainvillamide** | -7.309 | 1.181 | -3.970 | 1.635 | -5.66603 | 1.7276442 | -6.26027 | 1.4968921 |
| **25** | **Fiscpropionate D** | -9.747 | 1.030 | -6.635 | 1.57 | -6.19853 | 1.147398 | -7.68703 | 1.9602383 |
|  | **Ligand** | -5.532 | 1.635 |  |  | -5.96314 | 1.825157 |  |  |
|  | **Ampicillin** |  |  |  |  |  |  | -6.23181 | 1.65921 |
|  | **Desmopressin** |  |  | -6.757 | 1.421 |  |  |  |  |

**References:**

1. Kimura, M. A simple method for estimating evolutionary rates of base substitutions through comparative studies of nucleotide sequences. *J. Mol. Evol.* **1980**, *16*, 111-120, doi:10.1007/bf01731581.

2. Stecher, G.; Tamura, K.; Kumar, S. Molecular Evolutionary Genetics Analysis (MEGA) for macOS. *Mol. Biol. Evol.* **2020**, *37*, 1237-1239, doi:10.1093/molbev/msz312.

3. Tamura, K.; Stecher, G.; Kumar, S. MEGA11: Molecular Evolutionary Genetics Analysis Version 11. **2021**, *38*, 3022-3027, doi:10.1093/molbev/msab120.

4. Nei M; Kumar S. *Molecular Evolution and Phylogenetics*; Oxford University Press: New York, 2000.
